# Three-dimensional spatiotemporal focusing of holographic patterns

**DOI:** 10.1038/ncomms11928

**Published:** 2016-06-16

**Authors:** Oscar Hernandez, Eirini Papagiakoumou, Dimitrii Tanese, Kevin Fidelin, Claire Wyart, Valentina Emiliani

**Affiliations:** 1 Wavefront-Engineering Microscopy Group, Neurophotonics Laboratory, CNRS UMR 8250, Paris Descartes University, UFR Biomédicale, 45 rue des Saints-Pères, 75270 Paris Cedex 06, France; 2 Institut national de la santé et de la recherche médicale (Inserm), France; 3 Institut du Cerveau et de la Moelle Épinière, UPMC, Inserm UMR S975, CNRS UMR 7225, Campus Hospitalier Pitié Salpêtrière, 47 building de l'Hôpital, 75013 Paris, France

## Abstract

Two-photon excitation with temporally focused pulses can be combined with phase-modulation approaches, such as computer-generated holography and generalized phase contrast, to efficiently distribute light into two-dimensional, axially confined, user-defined shapes. Adding lens-phase modulations to 2D-phase holograms enables remote axial pattern displacement as well as simultaneous pattern generation in multiple distinct planes. However, the axial confinement linearly degrades with lateral shape area in previous reports where axially shifted holographic shapes were not temporally focused. Here we report an optical system using two spatial light modulators to independently control transverse- and axial-target light distribution. This approach enables simultaneous axial translation of single or multiple spatiotemporally focused patterns across the sample volume while achieving the axial confinement of temporal focusing. We use the system's capability to photoconvert tens of Kaede-expressing neurons with single-cell resolution in live zebrafish larvae.

## Abstract (web summary)

Three-dimensional computer-generated holography cannot be implemented with temporal focusing. Here, Hernandez *et al*. use two spatial light modulators to control transverse- and axial-target light distribution, generating spatiotemporally focused patterns with uniform light distribution throughout the entire volume.

## Graphical abstract

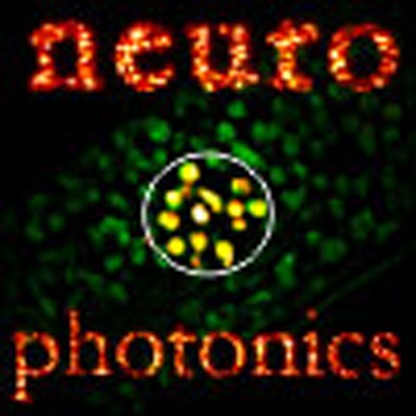

Since its first demonstration^1^, nonlinear two-photon (2P) microscopy has revolutionized diverse research fields including neuronal structural and functional imaging, photostimulation, laser processing and lithography. Original configurations were based on scanning a tightly focused pulsed laser beam across a defined sample region with galvanometric mirrors, acousto-optic deflectors^2^,^3^,^4^ or resonant scanners^5^. Recently, applications including lithography, uncaging, optogenetics and fast functional imaging have motivated scanless 2P-excitation method development, enabling simultaneous illumination of laterally extended regions of interest while preserving micrometre axial confinement. This was achieved by axially confining large spots generated by low-numerical aperture (NA) Gaussian beams with temporal focusing (TF)^6^,^7^. In TF, a diffraction grating conjugated to the sample plane diffracts the different spectral frequencies comprising an ultrashort excitation pulse towards different directions. The various frequencies thus propagate towards the objective focal plane at different angles, such that spatiotemporal coupling effects^8^ lead to temporal pulse broadening above and below the focal plane, which remains the only region irradiated at peak powers efficient for 2P excitation. In this way, TF enables axial confinement equivalent to that of line-scanning microscopy that is independent of the excitation beam's lateral extent. Investigators have applied TF to imaging^9^,^10^,^11^,^12^,^13^, functional imaging^14^,^15^, super-resolution imaging^16^, lithography^17^ and neuronal photostimulation^18^,^19^ by generating circular-spot shapes only.

As a more flexible way to elaborate light patterning, phase-modulation approaches using liquid-crystal spatial light modulators, such as computer-generated holography (CGH)^20^,^21^ and generalized phase contrast (GPC)^22^ have demonstrated efficient light sculpting in two-dimensional (2D) user-defined shapes. Combined with TF to axially confine the shapes, CGH and GPC can sculpt 2P-excitation volumes with the micrometre precision needed to illuminate small structures, such as neuronal dendrites^22^,^23^. These methods also enable 2P optogenetic stimulation with high temporal resolution and robustness to scattering^23^,^24^.

CGH can also sculpt light in three dimensions (3D)^25^, a feature used to generate multidiffraction-limited traps for optical tweezers^26^,^27^ and 3D glutamate uncaging^28^,^29^. Although not yet demonstrated with 2P excitation, adding lens-phase modulations to 2D-phase holograms also enables remote axial displacement and 3D positioning of laterally shaped targets^27^,^30^,^31^.

Combining CGH's 3D light-shaping capability with 2P excitation can significantly broaden the range of possible applications. For example, simultaneous multiplane holographic pattern generation can enable fast 2P multiplane imaging, photostimulation and photo-polymerization methods. In addition, remote axial displacement of holographic targets can couple holographic illumination with a second imaging or stimulation channel, providing independent control of their respective focal planes^32^, as well as remote volume scanning. However, previous 3D CGH optical configurations could not be implemented with TF because axially shifted excitation planes cannot be simultaneously imaged on the TF grating. This shortcoming restricted CGH-TF to 2D patterns focused at the objective focal plane^21^.

Here we demonstrate a unique optical system overcoming this limitation. The system achieves remote axial displacement of temporally focused holographic beams as well as multiple temporally focused planes by shaping the incoming wavefront in two steps using two spatial light modulators (SLMs). A first SLM laterally shapes the target light distribution that is focused on the TF grating, while a second SLM, positioned after the grating, controls the axial position(s) of the spatiotemporal focal plane(s) in the sample volume. By integrating phase profiles that minimize optical aberrations and intensity compensation protocols, we generate spatiotemporally focused patterns with uniform light distribution throughout the entire accessible volume, and demonstrate generation of laterally shaped targets across an unprecedented axial range. We apply axially confined multiplane light illumination to photoconvert *in vivo* Kaede-protein-expressing neurons in zebrafish larvae. The system enables cellular resolution photoconversion of tens of spinal cord neurons occupying separate axial planes.

## Results

### Optical system

**Figure 1:**
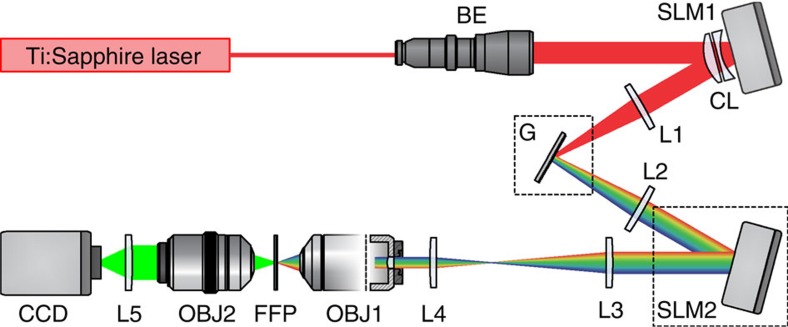
Experimental set-up for 3D-CGH-TF. The output beam of a Ti:Sapphire laser is magnified using a beam expander (BE) and projected on a first SLM (SLM1). SLM1 modulates the beam's phase so that light forms a user-defined intensity pattern on the diffraction grating (G) after passing through lens L1. The first diffraction order is collimated by the lens L2 and directed on a second SLM (SLM2). SLM2 imprints a lens-phase modulation that enables precise axial positioning of the spatiotemporal focal plane. The laser beam is then relayed and scaled by lenses L3 and L4 to the excitation objective (OBJ1) pupil size. OBJ1 is mounted on a piezo positioner so that it focuses and axially scans the excitation beam across a thin fluorescent layer. A second objective (OBJ2), always focused on the fluorescent layer, collects emitted fluorescence and forms an image on a CCD camera. Two cross-oriented cylindrical lenses (CL), with focal lengths of equal power and opposite sign, are used to suppress the zero-order spot of the first SLM^64^.

In the original optical system we designed (Fig. 1, see Methods), the output of a Ti:Sapphire laser was expanded to illuminate a first liquid crystal on silicon SLM (LCOS-SLM; SLM1) used to generate 2D holographic light patterns at the focal plane of a first lens where a blazed grating (G) was placed for TF. TF enables 2D CGH pattern generation with the axial confinement of a line-scanning microscope, practically decoupling axial confinement from lateral extent^21^ (Supplementary Fig. 1). Two telescopes (L2, L3; L4, OBJ1) relayed the holographic pattern on the sample plane. The phase holograms addressed on SLM1 were calculated using a Gerchberg and Saxton (GS)-based algorithm^33^. A second LCOS-SLM (SLM2) was placed at the confocal plane of the first telescope and imaged at the back focal plane of the microscope objective through the second telescope. By addressing SLM2 with single or multiple lens phases, we axially displaced single or multiple spatiotemporally focused patterns across the sample volume.

In order to characterize the optical properties of the holographic patterns, we used a microscope configuration using two opposite-facing objectives^20^,^21^. By doing so, holographic patterns illuminated a thin fluorescent film and 2P-excited fluorescence was collected by a second objective (OBJ2) placed opposite to OBJ1 and imaged on a CCD (charged couple device) camera. For 3D reconstruction of illumination volumes, OBJ2 was fixed and focused on the fluorescent layer, while OBJ1 was moved along the axial direction with a piezo-scanning stage^20^,^21^. For experiments on characterizing SLM's diffraction efficiency, the holographic patterns were directly imaged on the CCD camera (image of the transmitted laser light). For the experiments of photoconversion and optogenetic stimulation in zebrafish larvae, the system was coupled to a HiLo imaging set-up (see following sections).

The optical system enabled remote axial displacement of temporally focused holographic patterns as well as generation of multiple spatiotemporally focused holographic targets in distinct axial planes. In a simplified configuration where mirrors replaced the SLM2 and the grating, the system also generated 2P multiplane arbitrarily shaped non-temporally focused patterns.

### Remote axial shift of spatiotemporally focused patterns

First, we demonstrated remote axial displacement of a temporally focused holographic pattern by separating input-beam-phase modulation in two steps, controlling first the target lateral light distribution, and, second, its axial position. In contrast to conventional CGH, axial displacement of temporally focused holographic patterns cannot be achieved by simply adding a Fresnel lens to the phase hologram^34^ because the lens effect, by axially displacing the excitation plane with respect to the TF grating, would forbid the spatial and temporal focal planes to coincide^35^. We resolved this issue by implementing a novel, two-SLM strategy. SLM1 generated 2D CGH illumination patterns focused on the grating G, which dispersed the spectral components of the illumination pattern on SLM2. SLM2, conjugated to the objective back focal plane, was addressed with a Fresnel lens-phase profile to control the target's axial position in the sample volume. This design enables the spatial and temporal focal planes to coincide at the grating, and to be jointly translated by SLM2 across the sample volume axial extent.

The effect of spherical aberrations on targets generated out of OBJ1's nominal focal plane (Supplementary Fig. 2) was minimized by describing the objective focal sphere^36^ within the approximation of small defocus and high NA. We thus addressed SLM2 with lens-phase profiles featuring spherical phase^28^,^37^:


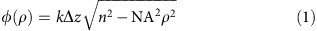


where *k* is the free-space wavenumber, *n* is the refractive index of the immersion medium, NA is the numerical aperture of the objective, 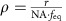 (with *f*_eq_ being the equivalent focal length: 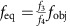 and 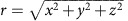) is the normalized pupil at the SLM2 plane and Δ*z* the axial displacement of the holographic light pattern in the sample volume.

**Figure 2:**
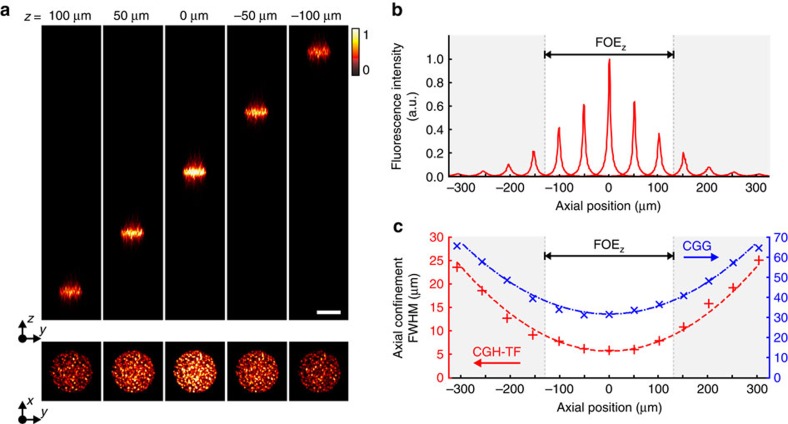
Axial displacement of spatiotemporally focused patterns. (**a**) Axial displacement of a 20-μm-diameter temporally focused holographic spot. Top, orthogonal maximum fluorescence intensity projection of the spot, for axial displacements of ±50 and±100 μm from the focal plane (0 μm). Bottom, corresponding *x–y* fluorescence intensity cross-sections. Scale bar, 20 μm. The colour bar refers to normalized intensity. (**b**) Axial profile of the integrated fluorescence intensity of the 20-μm-diameter holographic spot for different axial displacements. (**c**) Axial confinement (FWHM) of the profiles shown in **b** (CGH-TF; red data) compared with the axial confinement of a 20-μm-diameter holographic spot without TF (CGH; blue data). Data were fitted with a parabolic function (dashed lines) in both cases. White area in **b**,**c** represents the field of excitation (FOE_*z*_).

With the above strategy, we demonstrated remote axial translation of temporally focused holographic patterns by displacing a 20-μm-diameter holographic spot throughout a ±130-μm axial range (Fig. 2a–c), the accessible field of excitation (FOE_z_) without hologram aliasing (Supplementary Note 1). Within this range, we could remotely displace holographic patterns while conserving target shape sharpness (Fig. 2a, bottom) and axial confinement (5–10 μm; Fig. 2b,c). The integrated 2P fluorescence intensity decreased by approximately fivefold at the edges because of the diffraction efficiency of SLM2 (Supplementary Note 1 and Supplementary Fig. 3a–c).

Furthermore, we generated targets with micrometre axial confinement (10–25 μm) and sharp shapes across a broader axial range (±300 μm, that is, approximately two times greater than the FOE_z_). However, in this extended range, the full-width at half-maximum (FWHM) broadened (∼25 μm at ±300 μm; Fig. 2b,c), fluorescence intensity decreased (∼10 times at ±300 μm) and spot quality deteriorated (Supplementary Fig. 4) as a consequence of optical aberrations, SLM2 diffraction efficiency and the finite dimensions of mirrors and lenses placed after SLM2 that can vignette beam edges for highly diverging/converging wavefronts, among other factors.

For applications requiring constant illumination across the axial range, attenuating illumination intensity at axial positions near the centre of the FOE can compensate the position-dependent intensity decrease (Supplementary Fig. 3g). This can be accomplished either by reducing the incident laser power or by redirecting light into an extra spot similar to the procedure described in Supplementary Fig. 3d–f.

### 3D-CGH-TF spatiotemporally focused patterns

**Figure 3:**
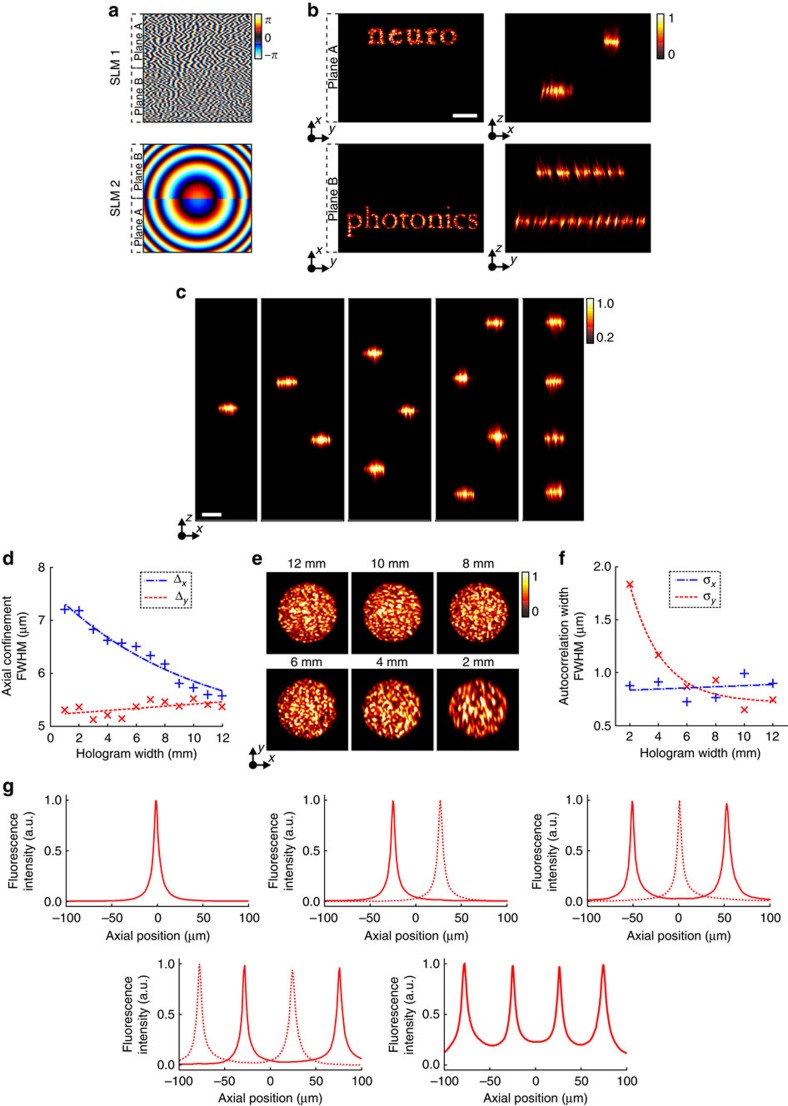
Multiplane spatiotemporally focused pattern generation. (**a**) Top, tiled phase profiles addressed to SLM1 for encoding the words ‘neuro' (plane A) and ‘photonics' (plane B). Bottom, Fresnel lens-phase profiles addressed to SLM2 to axially displace each holographic pattern generated by SLM1 on separated planes at +20 μm (plane A) and −20 μm (plane B). (**b**) Left, *x–y* 2P fluorescence intensity cross-sections at planes A and B generated by the holograms in **a**. Right, orthogonal maximum 2P intensity projection along *x* (top) and *y* (bottom). (**c**) Orthogonal fluorescence intensity projection of spatiotemporally focused patterns created by projecting 20-μm-diameter holographic spots in one, two, three and four planes at positions laterally shifted and in four planes at positions axially aligned (from left to right). Scale bars, 20 μm. (**d**) Axial confinement (FWHM) of a 20-μm-diameter holographic spot for different hologram widths, tested in both directions, Δ_x_ (blue data) and Δ_y_ (red data) parallel and perpendicular to grating's dispersion, respectively. (**e**) *x–y* 2P fluorescence intensity cross-sections of a 20-μm-diameter holographic spot generated with different hologram widths Δ_y_. (**f**) Autocorrelation width at the sample plane of a 20-μm-diameter holographic spot as a function of the hologram width along the *x*- (blue data; σ_*x*_) and *y* directions (red data; σ_*y*_). (**g**) Axial profile of the integrated fluorescence intensity of the holographic spots shown on **c**. Colour bars refer to normalized intensity.

Next, we demonstrated that decoupling lateral and axial light shaping into two separate steps also enables generation of multiplane spatiotemporally focused patterns. In this configuration, SLM1 is tiled into *n* vertical regions (that is, parallel to the orientation of the grating lines and orthogonal to the grating linear dispersion), with *n* equal to the number of axial planes populated by the final illumination pattern. Each tile generates a 2D target shape focused through L1 on the TF grating (Fig. 3a, top, Supplementary Fig. 5). SLM2, vertically tiled into *n* independent lens-phase profiles, then axially displaces each shape into its target axial plane (Fig. 3a, bottom). In this configuration, for each of the *n* targeted planes, both spatial and temporal foci coincide at the grating, while the *n* phase-lens profiles addressed on SLM2 enable independent remote displacement of each shaped target in the sample volume (Fig. 3b,c).

In agreement with previous observations^38^, reducing the hologram width, Δ*y*, along the direction perpendicular to the grating's dispersion did not compromise spot axial confinement, as the reduction of the hologram width, Δ*x*, in the orthogonal direction would do (Fig. 3d). Moreover, tiling holograms along the *x* axis would create crosstalk between axially separated planes because of the dispersion at the grating that generates at SLM2 a lateral spatial overlapping of the holograms generated by SLM1, making impossible to imprint independent axial shifts to the corresponding patterns.

However, vertical hologram resizing introduced a spatial filter that vertically elongated speckles (Fig. 3e). In particular, the vertical mean speckle size, estimated by calculating the vertical autocorrelation width^39^, σ_y_, increased by a factor of two with respect to the original size (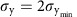) when the hologram vertical dimension was reduced to one-fourth of the full SLM aperture (Fig. 3f). In contrast, the horizontal autocorrelation width *σ*_x_ was unaffected by vertical hologram resizing (Fig. 3f). The limited size of the SLM can constitute the limiting factor defining the maximum number of axially separated planes in 3D-CGH-TF. Theoretically, the maximum number of planes is determined by the number of pixel rows on the SLM, but practically there is a tradeoff between the number of axial planes and the lateral resolution requirements for each application. As far as the spot shape is not distorted and spot's contour remains well defined, the smoothing of the excitation holographic spot to some extent can be even beneficial^21^,^38^,^40^.

Moreover, vertical SLM tiling induced a lateral tilt on the intensity propagation due to the asymmetric illumination of the objective back focal plane. Addressing SLM1 and SLM2 symmetrically along the vertical axis of the SLM eliminated this effect (Supplementary Fig. 6). Hologram resizing also affected the intensity of the illumination pattern at the sample plane. Specifically, holograms projected at the centre of the objective back aperture generated brighter targets than holograms projected on the side (Supplementary Fig. 7 and Supplementary Note 2). This effect adds to the position-dependent diffraction efficiency determined by the SLM pixel size (Supplementary Note 1 and Supplementary Fig. 3). Thus, generation of homogeneous 3D-CGH-TF patterns required compensating both lateral and axial position-dependent intensity variations, which in both cases was higher at the FOE centre and falling off towards the periphery. For lateral shape generation with SLM1, we weighted target intensity input to the GS algorithm such that targets occupying low-efficiency regions were brighter than targets position at the efficient FOE_*x,y*_ central zone (Supplementary Fig. 3). To achieve uniformity in the axial direction, we scaled the vertical tile size, allocating greater area to holograms projected at the objective pupil periphery with respect to the centre (Supplementary Note 2 and Supplementary Fig. 7). This enabled generation of homogeneous light patterns (Fig. 3g) across the whole excitation volume. Holographic patterns generated in distinct planes yielded fluorescence intensity distributions showing that axial confinement was also well conserved (Fig. 3g). However, an unavoidable background in the intermediate planes appeared when targets were laterally aligned (Fig. 3g, right bottom panel, and Supplementary Fig. 8).

### 3D computer-generated holography

**Figure 4:**
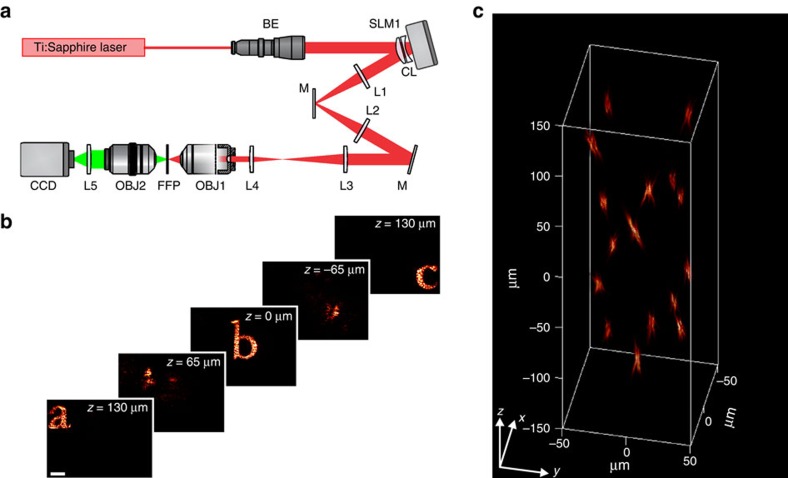
3D-CGH. (**a**) Schematic of the optical set-up for 3D-CGH. In this case the diffraction grating G and SLM2 were replaced by mirrors. (**b**) 2P fluorescence images of 3D-CGH patterns, depicting the letters ‘a', ‘b' and ‘c' at three different axial positions, *z*=130 μm, 0 and −130 μm, respectively. The phase profile used to project these patterns was calculated using a multiplane GS algorithm (Supplementary Fig. 9). Weighting of the input patterns according to their lateral and axial position (Supplementary Fig. 3) enabled diffraction efficiency correction and generated equal-intensity light patterns. (**c**) Volumetric reconstruction of three-dimensional distribution of 5-μm-diameter holographic spots (see also Supplementary Movie 1).

We demonstrate a simplified, single-SLM system for non-temporally focused multiplane pattern generation, replacing the grating and second SLM with mirrors (Fig. 4a); we patterned multiple targets occupying separate axial planes (Fig. 4b,c, Supplementary Movie 1) with a modified GS algorithm for multiplane pattern projection^41^ (Supplementary Fig. 9). By using the spherical phase expression described in equation (1) to translate the various targets into different axial planes, with 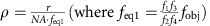, we minimized the effects of spherical aberrations on targets generated out of the focal plane, improving agreement between experimental and expected axial positions for the different targets compared with 3D-CGH implemented with parabolic-lens phases^42^ (Supplementary Fig. 2). We compensated diffraction efficiency-induced intensity variations by weighting target intensity inputs to the multiplane GS algorithm, as previously described (Supplementary Fig. 3), achieving uniform intensity shape generation throughout the accessible ∼240 × 240 × 260 μm^3^ volume (Supplementary Note 1).

### *In vivo* 3D-patterned selective photoconversion of neurons

**Figure 5:**
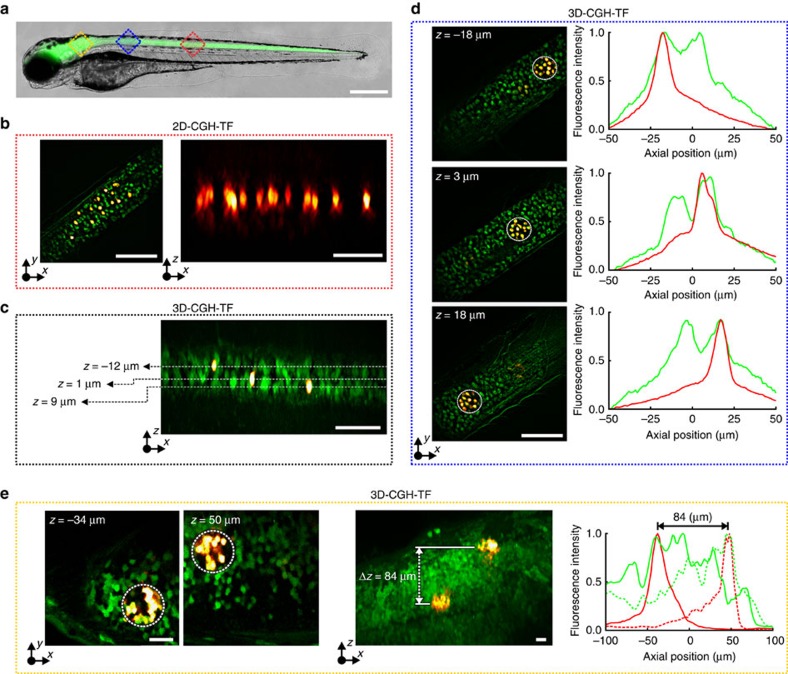
3D simultaneous 2P photoconversion of Kaede *in vivo*. (**a**) Merged brightfield and widefield fluorescence images of a double transgenic *Tg(HuC:gal4; UAS:kaede)* zebrafish larvae. Red and blue squares represent the approximate areas where we performed photoconversion. Scale bar, 400 μm. (**b**) Left, overlaid green and red HiLo fluorescence images before and after photoconversion, respectively. Right, orthogonal maximum red fluorescence intensity projection showing 14 photoconverted neurons on a single axial plane (illumination density 0.4 mW μm^−2^, 200 pulses of 50 ms). Scale bars, 60 μm. (**c**) Orthogonal maximum fluorescence intensity projection of overlaid HiLo pre- and post-photoconversion images (green and red fluorescence, respectively). Three single cells were photoconverted on separated axial planes (4.0 mW μm^−2^, one pulse of 200 ms). Scale bar, 60 μm. (**d**) Simultaneous 3D photoconversion of neural ensembles in the spinal cord. Left, overlaid HiLo pre- and post-photoconversion fluorescence images, where three 35-μm-diameter holographic spots projected at *z*=−18 μm, 3 and 18 μm were used for photoconversion (0.03 mW μm^−2^, 2,000 pulses of 50 ms). Right, axial distributions of green pre- and red post-photoconversion integrated fluorescence intensity over *z* for the spots projected at the three different planes. Scale bars, 60 μm. (**e**) Simultaneous 3D photoconversion of neural ensembles in the zebrafish brain. Left, overlaid 2P-excited green- and red post-photoconversion fluorescence images, where two 35-μm-diameter holographic spots projected at *z*=−38 μm and 50 μm were used for photoconversion (0.11 mW μm^−2^, 9,000 pulses of 50 ms). Scale bar, 20 μm. Middle, Orthogonal maximum 2P-excited fluorescence intensity projection of overlaid green and red-post-photoconversion images. Scale bar, 20 μm. Right, axial distributions of green pre- and red post-photoconversion integrated 2P fluorescence intensity over *z* for the spot at *z*=−38 μm (solid lines) and the one at *z*=50 μm (dotted lines). *z*-values in all cases are given as distances from the focal plane of the objective, which for the spinal cord experiments was at ∼60 μm and for the brain at ∼90 μm from the fish surface (where green fluorescence was starting). Positive *z*-values are closer to the surface. *λ*_phot_=800 nm.

We leveraged the axial specificity achievable with 3D-CGH-TF to photoconvert Kaede protein-expressing neurons in live *Tg(HuC:Gal4; UAS:Kaede)* double transgenic zebrafish larvae^43^. Kaede is a green photoactivable fluorescent protein that, when exposed to ultraviolet light, undergoes a photo-induced protein cleavage, red shifting its fluorescence emission spectrum^44^. With 2P excitation, efficient Kaede photoconversion can be obtained for wavelengths in the range of 760–800 nm^45^. We first performed Kaede photoconversion in spinal neurons (Fig. 5a, blue and red areas) to demonstrate the ability for single-cell resolution. Next, we demonstrated the robustness of 3D-CGH-TF to scattering by moving to the brain (Fig. 5a, dark yellow area) that is a more scattering region (Supplementary Fig. 10).

To monitor photoconversion in the spinal cord, we combined multiplane patterned photostimulation with a two-colour HiLo (high/low-frequency sequential acquisition) imaging system^46^ (see Methods and Supplementary Fig. 11). First, a HiLo *z*-stack exciting green fluorescence (excitation wavelength *λ*_exc_=473 nm) was acquired to localize Kaede-expressing neurons from which we selected photoconversion targets. We then photoconverted selected cells with 2P holographically patterned illumination (stimulation time 0.20–100 s; excitation power density: 0.03–4.0 mW μm^−2^; Methods. We note that power densities are always given relatively to the area of the spots' surface). A second HiLo *z*-stack exciting red fluorescence (*λ*_exc_=561 nm) was acquired after the photostimulation protocol in order to assess the photoconversion efficiency. For photoconversion experiments in the brain, scattering quickly deteriorates the quality of HiLo images. We therefore monitored the extent of photoconversion by moving the sample to a 2P galvo-based scanning imaging system in another set-up (see Methods). Green and red fluorescence in the 2P imaging system were excited at 780 nm, and scanning was performed at 0.74 Hz.

In the spinal cord, we first used 2D-CGH-TF to demonstrate simultaneous photoconversion (*λ*_phot_=800 nm) of multiple neurons in a single plane with cellular precision (Fig. 5a,b). Then, we used 3D-CGH-TF to photoconvert isolated neurons (Fig. 5c) or groups of multiple neurons (Fig. 5d) in distinct axial planes. The axial confinement achievable with 3D-CGH-TF enabled single-layer selectivity that would otherwise not be possible using CGH deprived of TF (Supplementary Fig. 12a). In all experiments performed, photoconversion induced a ∼10-fold increase in the ratio of pre- and post-photoconversion red fluorescence.

To demonstrate in-depth photoconversion, we performed a second set of experiments in the brain of zebrafish larvae by photoconverting neuronal ensembles in two different axial planes using 35-μm-diameter temporally focused holographic spots separated by ∼80 μm (Fig. 5e), with the first plane positioned at ∼90 μm from the surface. Although the axial confinement deteriorates with depth because of scattering, the red fluorescence intensity profiles along the axial direction showed selective and efficient photoconversion (Fig. 5d,e), which was not achievable with 3D-CGH alone (Supplementary Fig. 12b). Strikingly, axial resolution and overall shape were also preserved despite light propagation through the zebrafish brain (Supplementary Fig. 10). In agreement with previous results in rats^23^,^24^, these results demonstrate that TF enables robust in-depth propagation of shaped patterns through scattering media.

It is important to note that 2P Kaede photoconversion is a low-efficiency process and requires relatively high illumination doses (∼0.04–4 mW μm^−2^) and long (from 200 ms to a few hundreds of seconds) stimulation protocols. Other type of applications such as activation of neurons via 2P-mediated optogenetic stimulation can work with drastically reduced photostimulation time (5 pulses of 50 ms), as well as lower illumination doses (0.04–0.60 mW μm^−2^; Supplementary Note 3).

A still open question for multiplane multispot photostimulation concerns the possible temperature rise induced by laser illumination. For the illumination doses we used in the optogenetics experiments (Supplementary Note 3), we estimated that this does not exceed few degrees Celsius. Specifically, by solving the heat diffusion equation^47^ with typical thermal optical parameters of tissue found in literature^48^,^49^,^50^, we estimated the mean temperature rise to be of the order of 0.06–0.25 °C, using illumination pulses of 50 ms in the range of 0.04–0.6 mW μm^−2^ and a 10-μm diameter spot (Picot *et al.*, private communication). Using 10 spots of the same size distributed randomly over a FOE of 100 × 100 μm^2^ would generate a mean temperature rise of 0.9–3.7 °C. Diffusion over the tissue volume enables the temperature to return to its equilibrium value in less than 150 ms, which was the interval between illumination pulses (Picot *et al.*, private communication).

## Discussion

We have demonstrated a unique optical system enabling remote axial displacement of temporally focused holographic patterns, as well as generation of multiple temporally focused holographic targets occupying separate axial planes. In our two-step system, the first SLM is addressed with phase holograms controlling the transverse target light distribution. The second SLM, positioned after the TF grating, is addressed with Fresnel lens-phase functions and controls target axial position. We demonstrated that this configuration can jointly translate single or multiple spatiotemporally focused patterns across the sample volume.

We demonstrated axial displacement of a single temporally focused holographic pattern across an axial range (±300 μm), roughly two times greater than the nominal accessible axial FOE, FOE_z_ (±130 μm). For axial shifts of 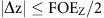, spot shape and axial resolution (5–10 μm) were well conserved, while fluorescence intensity decreased approximately fivefold (consistent with the roughly twofold decrease in illumination intensity, Supplementary Fig. 3). Displacements up to twice the accessible axial FOE were also possible, although requiring compensation mechanisms to correct for broadening of axial confinement (∼25 μm for Δ*z*=±300 μm) and intensity losses.

With our approach, applications requiring continuous fast scanning of a single spatiotemporally focused target will be limited by the SLM refresh rate (60–200 Hz). Replacing SLM2 with a tunable lens^51^ would overcome this limitation. However, it would not enable generation of multiple spatiotemporally focused targets at distinct axial planes.

Alternative solutions for axial displacement of a temporally focused shape used either variable group velocity dispersion (GVD)^38^,^52^,^53^,^54^,^55^ or mechanical axial translation of the TF grating^35^. Indeed, introducing variable GVD to the input laser pulse enabled efficient axial displacement of a Gaussian beam's temporal focal plane. However, GVD-induced axial displacement strongly depends on the autocorrelation width of the illumination patterns, which for holographic spots is small due to speckles. Therefore, in order to achieve axial displacement of temporally focused holographic patterns, spatial filtering is required to increase the correlation width. However, this degrades spatial resolution and decreases illumination intensity^38^. Furthermore, the GVD approach achieved axial displacements of only a few microns^38^. Greater scanning ranges could be obtained by combining 2D-CGH with the optical design proposed by Dana *et al.*^35^ for axial scanning of a temporally focused line. The system implemented on-axis-light propagation and mechanical axial translation of the TF grating. However, for high-magnification (M) objectives, the axial shift, *d*, was limited by the long required grating translation*, D*∝*d* × *M*^2^. Thus, the maximum shift achieved with a × 40 objective was inferior to 30 μm.

In addition to limited range, neither GVD shift nor grating translation enable the generation of multiple temporally focused targets at distinct axial planes. Here we overcome this limitation with a two-step, two-SLM system, addressing a first SLM with multiple vertically tiled phase holograms, each encoding the light distribution of a single plane, and a second SLM with an equal number of Fresnel lens phases, which individually control the axial position for each plane. In this way, all targets are projected to the TF plane, which is kept at a fixed position, while the second SLM imposes the spatial wavefront curvature needed to displace each plane axially.

The number of spatiotemporal focal planes that can be generated with this design is a tradeoff between the number of available pixels and spatial resolution. Here we demonstrated generation of up to four spatiotemporal focal planes. In this case, the vertical spatial resolution (that is, the average speckle size in the *y* axis), where tiling occurs, decreases roughly twofold because of the reduced size of the hologram at the objective back aperture. LCOS-SLM devices with increased number of pixels should increase the number of achievable planes and reduce hologram resolution deterioration.

Moreover, we implemented a simpler single-SLM system to generate multiplane CGH light patterns without TF. Although this approach has a poor axial confinement with respect to 3D-CGH-TF, it could still be a powerful method for applications using sparsely expressing samples. Previous schemes for multiplane generation of laterally extended shapes through high-NA objectives reported patterns spanning a limited (tens of microns) axial range^27^,^30^,^31^ and used linear excitation. Here we exploited the whole excitation volume reachable with the LCOS-SLM using high-NA objectives. This was achieved by minimizing spherical aberration of targets generated out of the nominal objective focal plane by accounting for the high-NA objective in the expression of the Fresnel lens-phase profile. In addition, throughout the accessible volume, we obtained uniform light distribution among the target shapes. Weighting the amplitude of each target input to the GS algorithm according to position enabled compensation of intensity loss because of diffraction efficiency, which decreases with increasing distance from the excitation volume centre.

We demonstrated the capabilities of the 3D-CGH-TF system by performing multiplane 2P photoconversion of Kaede-expressing neurons. Previous *in vivo* experiments photoconverting Kaede in single neurons of zebrafish larvae used one-photon visible light illumination^43^,^56^,^57^. Here we demonstrated 2P-mediated single-cell resolution simultaneous photoconversion of multiple Kaede-expressing neurons. The unprecedented spatial precision achieved by our approach can, in the future, be extended to track the morphology of single neurons across the entire nervous system of the zebrafish larva.

For multiplane photoconversion of multiple isolated targets (for instance, in sparsely expressing samples), 3D-CGH holds an advantage over 3D-CGH-TF by enabling light shaping in a greater number of planes. However, 3D-CGH-TF enables a better axial confinement, a key parameter for experiments requiring illumination of spatially nearby multiple targets or large areas. Moreover, generation of multiplane illumination patterns with 3D-CGH also produces spurious light in the intermediate planes absent in illumination geometries produced with 3D-CGH-TF. In addition, we have previously demonstrated that TF is particularly robust to scattering making 3D-CGH-TF more suitable than 3D-CGH for applications requiring in-depth illumination through scattering media^23^,^24^.

The same optical design could be used to photoswitch other proteins such as photactivatable green fluorescent protein^58^ or kindling fluorescent protein (KFP1)^59^ to precisely track the 3D position of specific cells *in vivo* during embryo development. Combined with optogenetics (Supplementary Note 3) or uncaging, multiplane generation of spatiotemporally focused patterns will enable simultaneous control of neurons and substructures in different planes, as well as provide a flexible mean to stimulate locations lying above or below the imaging plane. Combined with extended depth-of-field imaging^60^, multiplane light patterning could also improve the spatial specificity of functional voltage or calcium physiology by shaping light on cells or structures of interest^61^.

In this study, the two-SLM system is combined with HiLo imaging. Combining this approach with 2P imaging would also enable deep photostimulation and imaging *in vivo*. Decoupling of lateral and axial wavefront shaping could also be adopted in optical designs different from the one presented here. For example, placing a second SLM at the Fourier plane of a fast switchable array should enable 3D-encoded multisite 2P microscopy^62^ or high-speed 3D holographic light patterning^63^.

## Methods

### Two-SLM optical set-up

The optical system, schematically depicted in Fig. 1 and Supplementary Fig. 11, was built around a commercial Olympus IX71 inverted microscope, modified in order to accommodate two opposite-facing objectives, OBJ1 and OBJ2, for excitation and fluorescence collection, respectively. To this end, the condenser lens of the microscope was substituted with a dielectric mirror and an Olympus LUMPLFL60xW/IR2, NA 0.90 objective (OBJ1). The expanded (× 10) beam of a Ti:Sapphire laser (MaiTai Deep-See, Spectra-Physics) covered the active area of a first LCOS-SLM (X10468-07, Hamamatsu Photonics; SLM1), which modulated the phase of the incoming beam to create a first image of the desired intensity pattern on the diffraction grating (830 l/mm, 53004ZD02-035R, Richardson Gratings; G) for TF through the lens L1 (*f*_1_=500 mm). 2D-phase holograms were calculated using a standard GS algorithm^20^,^33^. The first diffraction order was subsequently collimated by lens L2 (*f*_2_=500 mm) and impinged on a second SLM (X10468-07, Hamamatsu Photonics; SLM2), which was imaged at the back focal plane of the excitation objective, OBJ1, via a 2:1 telescope (lenses L3, *f*_3_=1,000 mm and L4, *f*_4_=500 mm). Suppression of the zero-order spot arising from SLM1 was achieved by using two cylindrical lenses (*f*_L1_=1,000 mm and *f*_L2_=−1,000 mm) oriented at +45° and −45° with respect to the grating lines^64^.

Holographic light patterns generated at the sample volume illuminated a thin spin-coated fluorescent layer of rhodamine-6G in polymethyl methacrylate 2% w/v in chloroform and the induced fluorescence was imaged on a CCD camera (CoolSNAP HQ2, Roper Scientific) through OBJ2 (Olympus UPLSAPO60XW, NA 1.2). For 3D reconstruction of illumination volumes, OBJ2 was fixed and focused on the fluorescent layer, while OBJ1 was moved along the axial direction with a piezo positioner of 1 mm range when working in closed loop (PI N-725.2A PIFOC). The two SLMs, the CCD camera, the piezo positioner, lasers and other electronic components of the set-up were controlled by a custom-developed interface in LabVIEW. GS-based algorithms were run in MATLAB.

When the set-up was used for generation of multiplane holographic patterns (not temporally focused) a mirror replaced the diffraction grating, and SLM2 was either used in reflectance mode by applying only the flatness correction phase mask of the device or was replaced by a mirror. Both the grating and SLM2 were mounted on magnetic bases enabling fast switching between the different configurations. The multiplane GS algorithm used in this case was run in a custom-designed C++ software interface, Wavefront Designer^20^.

### Two-colour HiLo imaging system

High-resolution multiplane fluorescence imaging of zebrafish larvae was achieved by coupling an optical set-up for two-colour HiLo microscopy^46^ to the Olympus IX71 microscope. Two continuous wave 473-nm (Laser Quantum, Ciel 350 mW) and 561-nm (CNI laser, MGL-N-561-500 mW) lasers were co-aligned in the same optical path with the dichroic mirror D2 (Semrock, Di02-R514) and collimated with lenses L6 (*f*_6_=125 mm), L7 (*f*_7_=150 mm) and L10 (*f*_10_=35 mm) to illuminate an oscillating diffuser plate (Optotune LSR-3005-10) that was imaged through lenses L8 (*f*_8_=75 mm) and L9 (*f*_9_=200 mm) at the back aperture of the excitation objective, OBJ1 (Supplementary Fig. 11). The D1 dichroic mirror reflected the collected fluorescence to a CMOS camera (Hamamatsu Photonics, Orca Flash 4.0-V2) through the appropriate filter cube (FC2) for green (dichroic mirror Semrock FF495-Di02, emission filter Semrock FF01-520/35-25) or red fluorescence (dichroic mirror Semrock Di02-R561, emission filter Semrock FF595-Di02).

The sectioned image was computed with custom scripts written in MATLAB^65^. The cutoff frequency used to merge the low- and high-frequency components was chosen such as *k*_c_≈0.1 *k*_low_, where *k*_low_ is the frequency of the low-pass filter applied to the uniform illumination image. With those parameters, we measured an axial resolution of 3.2-μm FWHM for the emitted fluorescence (Supplementary Fig. 11b). The axial resolution was measured using a Rhodamine-6G thin layer. First, we recorded a *z*-stack with uniform illumination (oscillating diffuser on) and then with speckle illumination (oscillating diffuser off). The two stacks were then processed in MATLAB to generate an axially resolved HiLo *z*-stack using algorithms previously described^34^,^65^. The axial resolution shown in Supplementary Fig. 11 is the axial resolution measured on the processed HiLo *z*-stack.

### 2P galvo-based scanning imaging system

2P imaging of photoconverted zebrafish larvae in the brain performed by a mode-locked Ti-Sapphire laser source (Coherent Chameleon Vision II, pulse width 140 fs, tuning range 680–1,080 nm). The femtosecond pulsed beam was raster-scanned on the sample via a pair of *xy* galvanometric mirrors (3 mm aperture, 6215H series, Cambridge Technology) imaged at the back aperture of the microscope objective (× 40 W APO NIR, Nikon) through an afocal telescope (scan lens: *f*=100 mm, tube lens: *f*=300 mm). Galvanometric mirrors were driven by two servo drivers (MicroMax series 671, Cambridge Technology) controlled by a Digital/Analog converter board (PCI-6110, National Instrument). Emitted fluorescence was collected by a fibre-coupled detection scheme^66^. The fibre exit was imaged on two photomultiplier tubes GaAsP (H10770-40 SEL, Hamamatsu Photonics, active area 5 mm) by a set of three matching asphere lenses (*f*=23.5 mm, Melles Griot #LAG-32.5-23.5-C). Following the fibre exit, fluorescence light was filtered with an infrared-light-blocking filter (FF01-750sp, Semrock), split into two channels by a dichroic mirror (FF555-Di03, Semrock) and detected through two emission filters (FF01-510/84 and FF02-617/73, Semrock). The whole system was built around a commercial upright microscope (SliceScope, Scientifica). 2P imaging laser power was tuned by combining an electrically controlled liquid crystal variable phase retarder (LRC-200-IR1, Meadowlark Optics) and a polarizer cube (BB-050-IR1, Meadowlark Optics) at the exit of the laser source.

Green and red fluorescence *z*-stacks of photoconverted Kaede in the zebrafish brain were acquired by scanning the excitation beam (780 nm) at 0.74 Hz (full frame) and averaging 10–20 frames for each plane.

### Photoconversion protocol

First, a HiLo z-stack in the green channel (200 × 200 × 100 μm^3^) was recorded to map the location of neuronal cells for photoconversion. On the basis of these images, we calculated phase holograms that produced the corresponding 2D or 3D illumination patterns. We typically used 5-μm-diameter holographic spots to target single cells and 30–35-μm-diameter holographic spots to target sets of neurons. In order to quantify the efficiency of photoconversion, we also recorded the corresponding z-stack in the red channel before photoconversion.

Simultaneous 2P photoconversion (*λ*_phot_=800 nm) of all targets was performed while monitoring the fluorescence in the red channel. We typically observed a tenfold increase of red fluorescence in the targeted cells. Photoconversion during fluorescence imaging was minimized by keeping the total acquisition time below 2 min and laser power at the sample plane below 20 mW. To minimize thermal damage during photoconversion, we delivered trains of 50-ms pulses, low laser intensity ∼0.04–4.0 mW μm^−2^ (power densities are always given relatively to the area of the spots' surface) for periods of time that ranged from 200 ms to a few hundred seconds depending on the laser intensity.

### Transgenic lines

Experiments were performed on Danio rerio larvae between 2 and 6 days post fertilization following procedures approved by the Institutional Ethics Committee Darwin in the ‘Institut du Cerveau et de la Moelle épinière' (ICM). AB and TL strains of wild-type (WT) larvae were obtained from laboratory's stock of adults. Embryos and larvae were raised in an incubator at 28.5 °C until shortly before recordings were performed. For photoconversion experiments, we used *Tg(HuC:gal4; UAS:kaede)*^43^ where the *HuC* promoter drives pan-neuronal expression of Gal4 and Kaede at the larval stage. *Tg(pkd2l1:gal4; UAS:ChR2-H134R-mCherry; UAS:GCaMP5G)*^65^,^67^ were used for combination of optogenetics and calcium imaging in Supplementary Data. Before performing image acquisitions, embryos were dechorionated and screened for fluorescence at 1 days post fertilization. Larvae screened for Kaede fluorescence were later embedded laterally in 1.5% agarose. Larvae were anaesthetized in 0.02% tricain (MS-222, Sigma-Aldrich, USA).

### Data availability

The data that support the findings of this study are available from the corresponding author upon request.

## Additional information

**How to cite this article:** Hernandez, O. *et al.* Three-dimensional spatiotemporal focusing of holographic patterns. *Nat. Commun.* 7:11928 doi: 10.1038/ncomms11928 (2016).

## Supplementary Material

Supplementary Information Supplementary Figures 1-14, Supplementary Notes 1-3 and Supplementary References.

Supplementary Movie 1 3D-CGH, Volumetric reconstruction of three-dimensional distribution of 5-μm diameter holographic spots.
