# Erratum: Three-dimensional spatiotemporal focusing of holographic patterns

**DOI:** 10.1038/ncomms12716

**Published:** 2016-09-02

**Authors:** Oscar Hernandez, Eirini Papagiakoumou, Dimitrii Tanese, Kevin Fidelin, Claire Wyart, Valentina Emiliani

Nature Communications 7: Article number: 11928 10.1038/ncomms11928 (2016); Published: 06 016 2016; Updated: 09 02 2016

An incorrect version of the Supplementary Information was inadvertently published with this Article that contained errors in Supplementary Equations 18 and 19. The HTML has now been updated to include the correct version of the Supplementary Information.

