# Supplementary material for: Three-dimensional spatiotemporal focusing of holographic patterns
Source: Nat Commun. 2016 Jun 16;7:11928. doi: 10.1038/ncomms11928 (PMC4912686; doi:10.1038/ncomms11928)
Supplement: Supplementary Information — Supplementary Figures 1-14, Supplementary Notes 1-3 and Supplementary References. [file ncomms11928-s1.pdf]

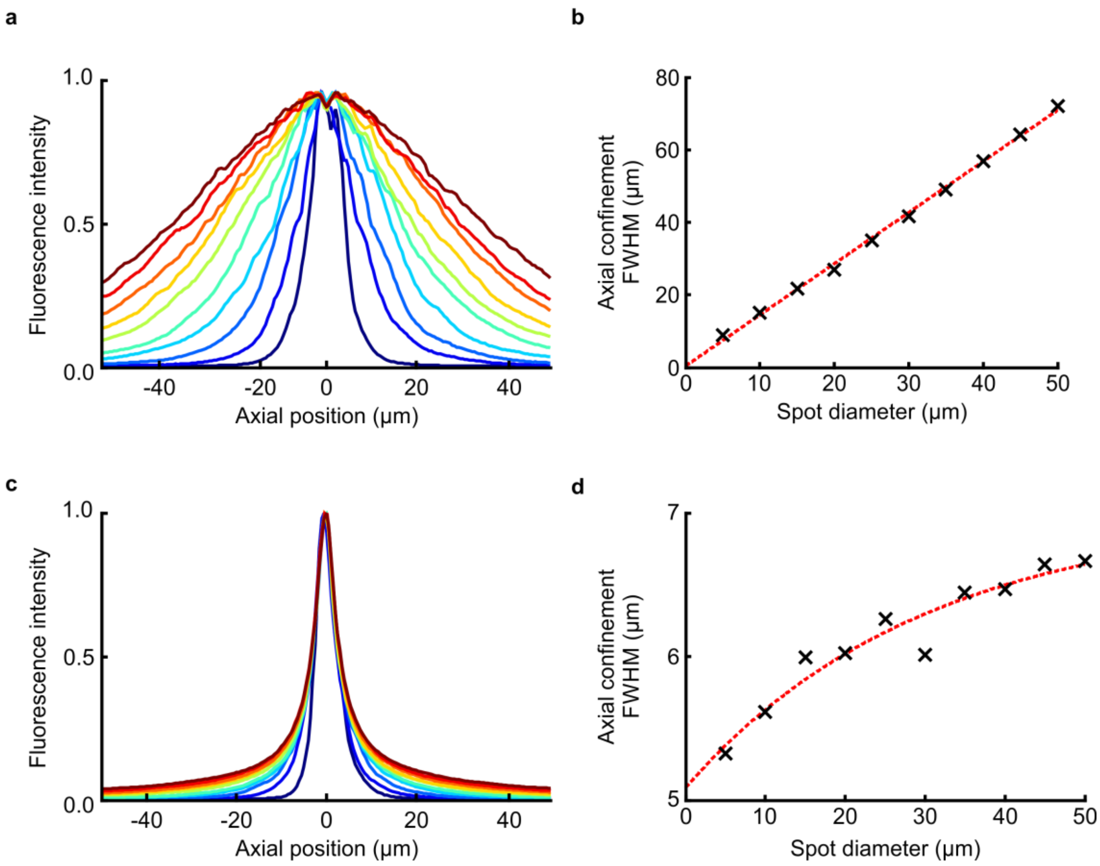

Supplementary Figure 1 | Axial confinement and lateral spot size of holographic spots. (**a**, **b**) Measured axial profiles (**a**), and axial confinement (FWHM) (**b**) of the 2P fluorescence intensity distribution produced by circular holographic spots of increasing size (5-50 µm in diameter). (**c**, **d**) Measured axial profiles (**c**) and, axial confinement (FWHM) (**d**) of the 2P fluorescence intensity distribution produced by temporally focused holographic spots of increasing size (5-50 µm in diameter). Excitation objective: 60x, 0.9 NA.

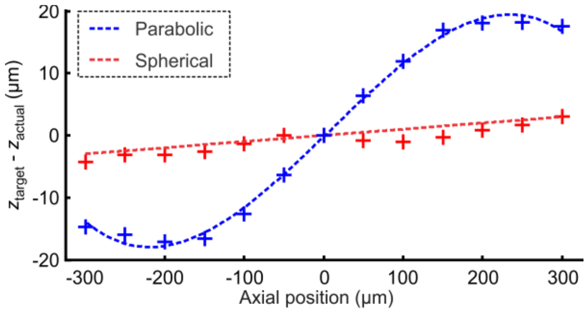

Supplementary Figure 2 │ Actual axial position of holographic spots. Difference between the expected and measured axial position of a 20-μm-diameter holographic spot as a function of the axial displacement. Blue curve and data show the parabolic case, where the paraxial approximation was used for calculating the Fresnel-lens phase profile for the axial displacement. Red curve and data show the case where a spherical phase description was used for the Fresnel-lens phase profile, minimizing thus spherical aberrations.

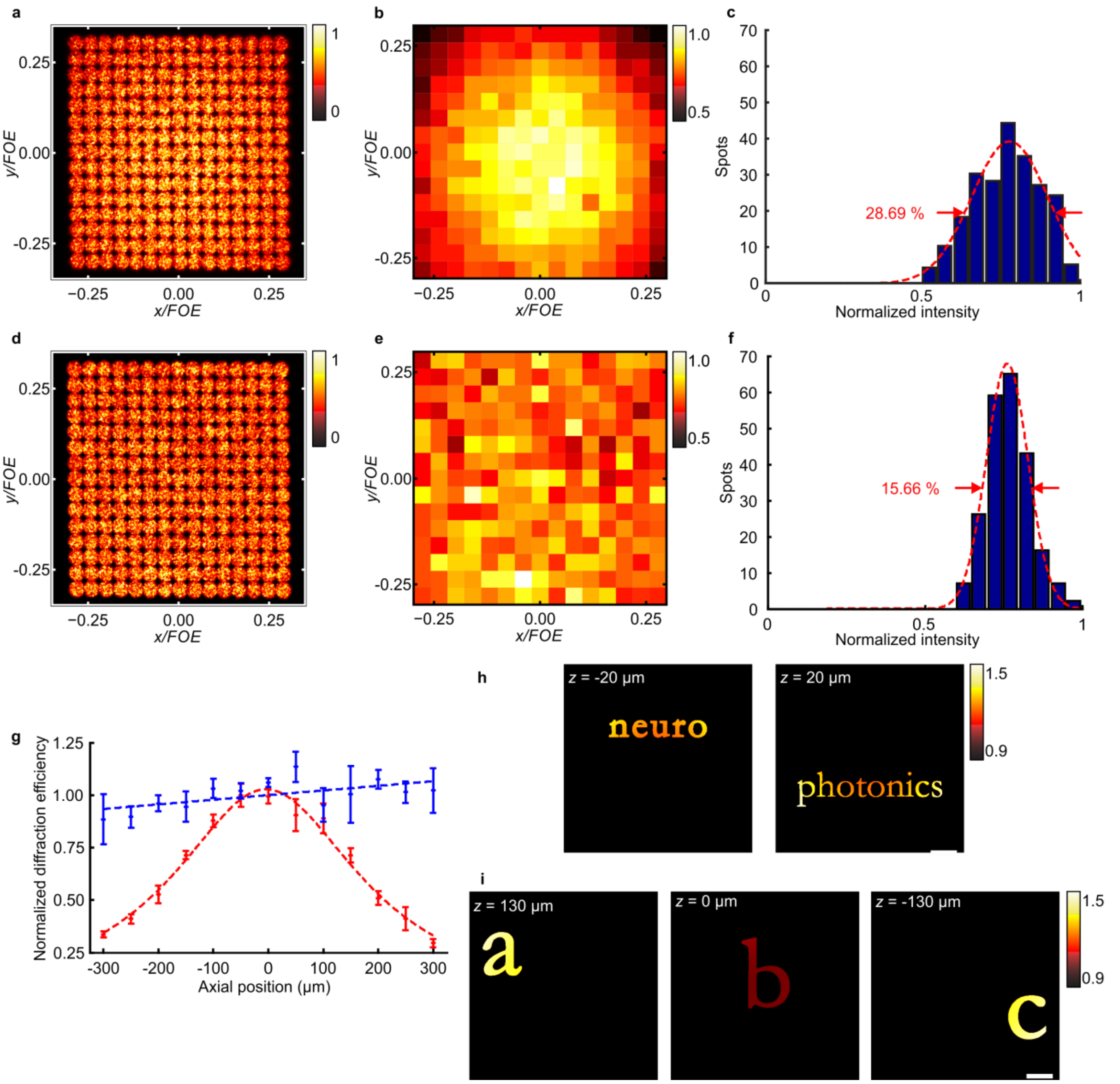

Supplementary Figure 3 | SLM diffraction efficiency characterization and correction. (**a**) Maximum intensity projection of 10-μm-diameter holographic spots projected individually in positions forming a 15 x 15 grid. (**b**) Map of the diffraction efficiency for each spot in **a**, normalized to the one of the central spot. Each pixel corresponds to one spot. (**c**) Histogram of the normalized integrated intensity of the spots. (**d**) Same as in **a** after intensity compensation**.** In this case, homogenization of light distribution was achieved by projecting 4 extra spots out of the camera field of view (~168 × 224 μm^2), enabling to keep the spot intensity constant for every position in the grid. The 4 extra spots can be blocked by adding a diaphragm in an intermediate imaging plane of the optical system, conjugated to the sample plane. Note that x/FOE and y/FOE are unit-less numbers indicating the position of each point relatively to the FOE. (**e**) Map of the compensated lateral diffraction efficiency. (**f**) Histogram of the normalized integrated intensity of the compensated spots showing a two-fold decrease in the spots' intensity distribution width. (**g**) Axial diffraction efficiency curve, where the intensity of each holographic spot projected at each axial plane is normalized to the one of a spot projected at the center of the nominal focal plane. Experimental

data (red points) represent the average of four realizations and follow a Lorentzian distribution (red dashed line) with Δz_{FWHM}=360 µm. Weighting the axially displaced holographic spot intensity according to the calculated diffraction efficiency enables intensity equalization between holographic patterns in separated axial positions (blue points and dashed line represent the corrected intensity ratio and fitting, respectively). Vertical error bars show the standard deviation for the different realizations. (**h-i**) Input patterns to the GS algorithm used to calculate the holograms that generate the holographic patterns shown in Fig. 3b (**h**) and Fig. 4b (**i**)**:** low-diffraction efficiency regions appear brighter over those closer to the center of the excitation field. To improve the observation of differences between conditions, the amplitude scale of the images was chosen from 0.9 to 1.5.

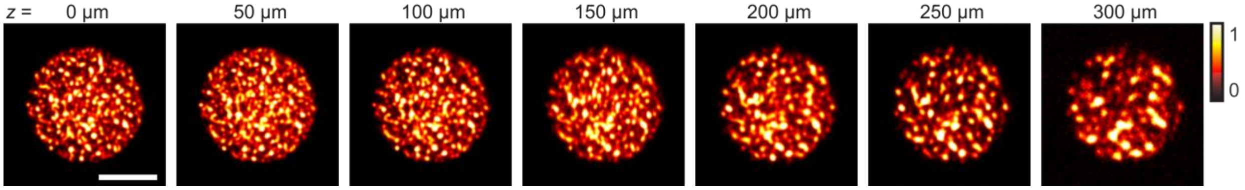

Supplementary Figure 4 | Remote axial displacement of a temporally focused holographic spot: x-y cross sections of a 20-µm-diameter holographic temporally focused spot displaced across an axial range of 300 µm. The shape of the spot remains unchanged for axial displacements within the aliasing free range ($|\Delta z|$= 130 µm). For $|\Delta z|$>130 µm the spot uniformity starts to deteriorate and speckle-size increases due to aliasing in the lens profile and, therefore, a reduction of the effective objective numerical aperture. Scale bar: 10 µm.

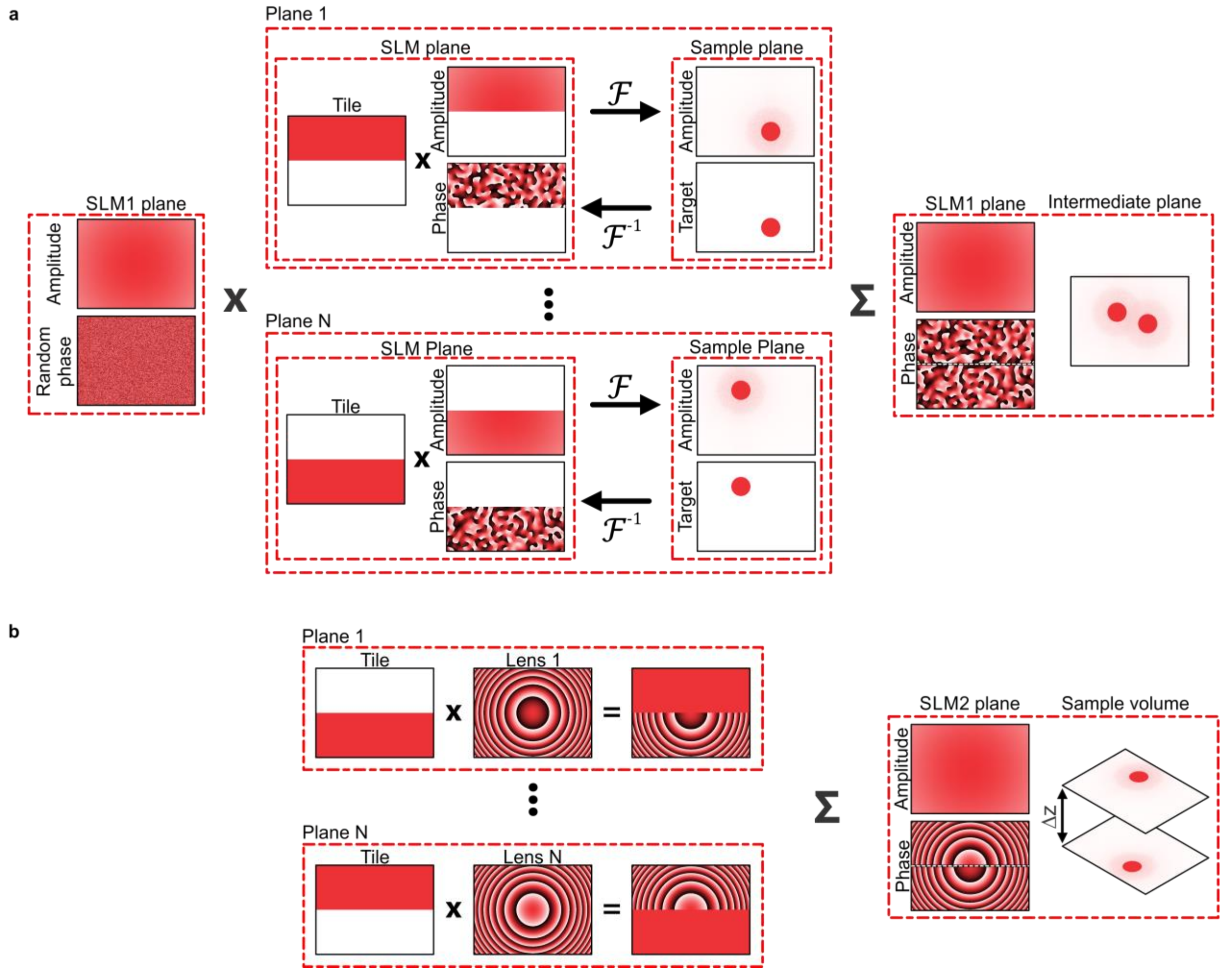

Supplementary Figure 5 | 3D-CGH-TF algorithm scheme. (**a**) SLM1 illumination. SLM1 is tiled into n vertically regions equivalent to the number of axial planes in the final 3D pattern. Each of these regions is addressed with an independent phase-hologram calculated with a modified Gerchberg and Saxton (GS) algorithm[1], where we added a memory writing operation (or multiplication), in which we set the electric field to zero outside of each tile. In this way, we obtain distinct phase profiles each encoding for a distinct holographic pattern. Right, the final phase profile addressed to SLM1 is a vertical superposition of the distinct ones. (**b**) SLM2 illumination. Left, SLM2 is addressed with vertically tiled cropped Fresnel-lens phase profiles, each controlling the axial position of the conjugated holograms at SLM1. Right, the final phase profile addressed to SLM2 is a vertical superposition of the distinct Fresnel-lens phase profiles. As a result, different targets are displaced at distinct axial planes.

The computation time of our algorithm can be estimated in the basis of the conventional GS, where the total computation time increases linearly with the number of separated axial planes. The latter varies drastically depending on the computer characteristics and the implementation of the algorithm. In our case, since our application did not critically depend on fast kinetics, we did not take special care to reduce the computation time to the minimum possible value. Typical computation time in our system for 4 planes was a few seconds (< 5 s). Optimal implementations of these algorithms in graphics processing units could reduce the calculation time down to few milliseconds[2].

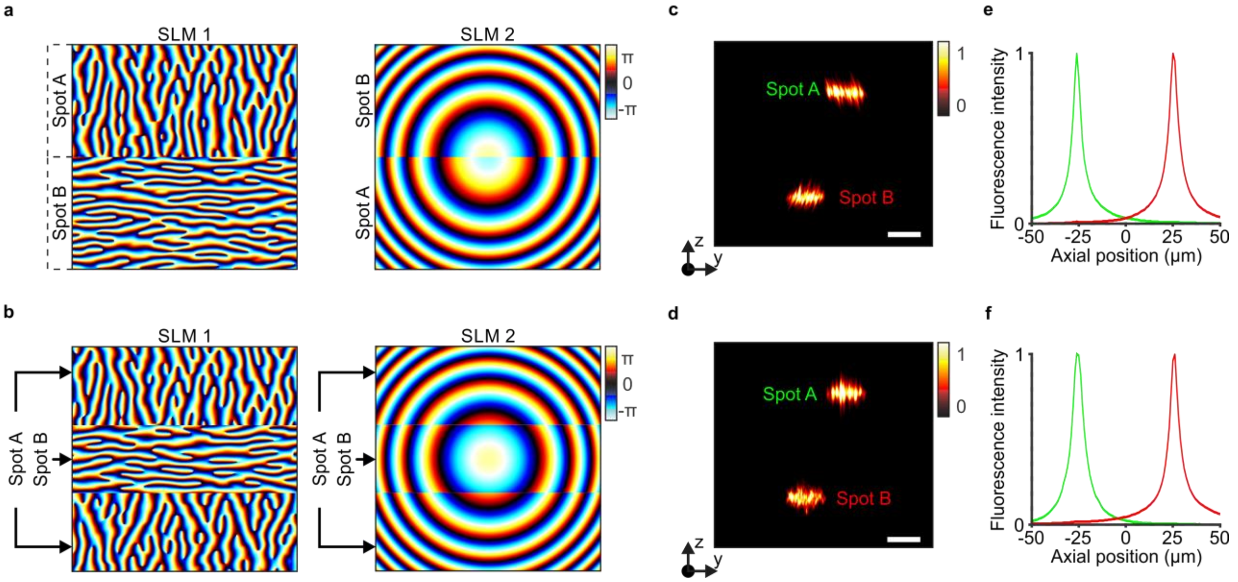

Supplementary Figure 6 │ Tilt correction using symmetric tiling. (**a-b**) Tiled phase profiles addressed to SLM1 and SLM2 encoding two 15-μm-diameter spots axially displaced by -25 μm (spot A) and +25 μm (spot B) from the objective focal plane. Asymmetric tiling (**a**) introduces a tilt on the propagation (**c**), as it implies an asymmetric illumination of the objective back aperture for each phase profile. Even though this tilt has no significant impact on the axial resolution of the spots (**e**) we can correct it (**d**) by using symmetric tiling of the phase profile for each plane, along the vertical axis of the SLM (**b**). (**c-d**) Orthogonal maximum fluorescence intensity projection and (**e-f**) axial distribution of the integrated fluorescence intensity for each case (**a**) and (**b**), respectively. Scale bars: 15 μm.

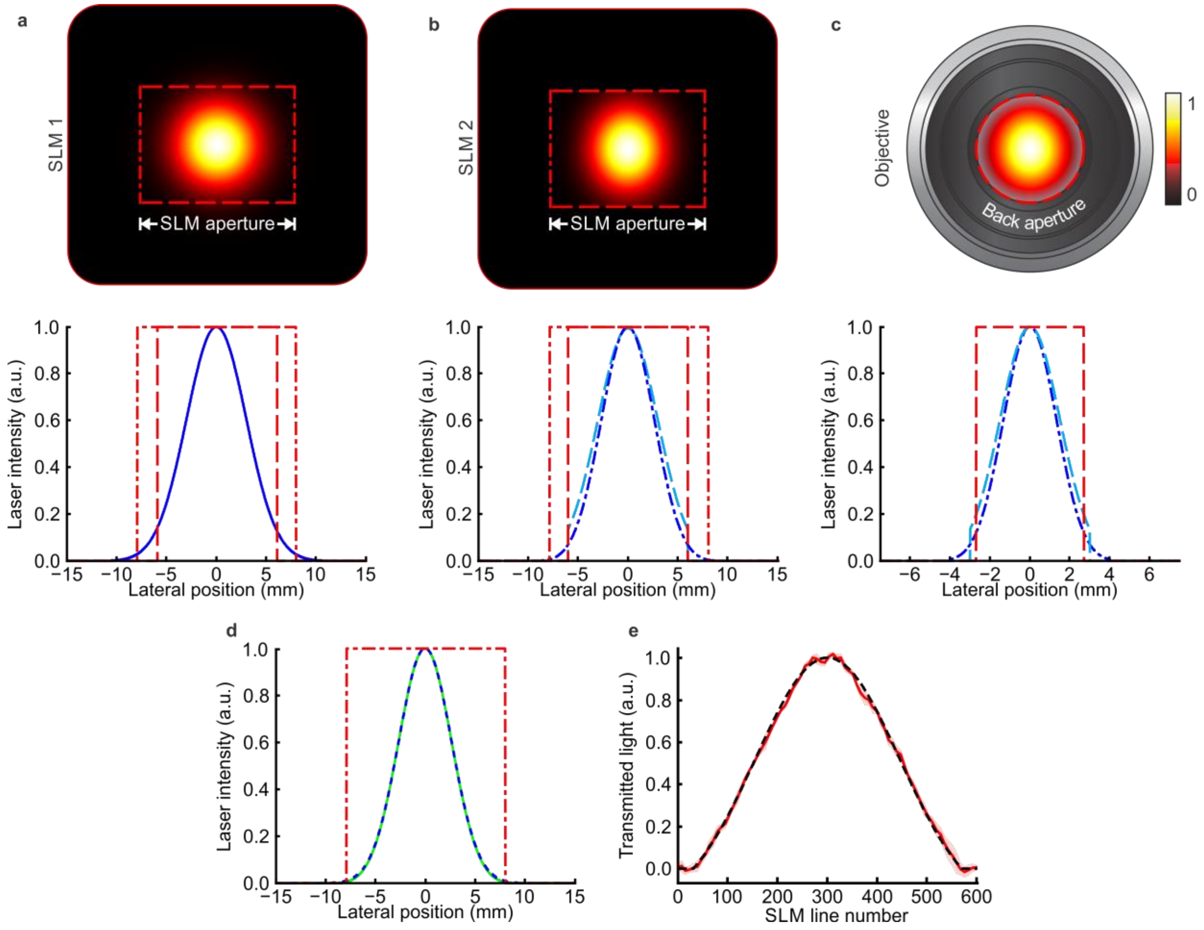

Supplementary Figure 7 │ Light propagation through the optical setup. (**a-c**) Top, x-y intensity distribution on, **a**, SLM1, **b**, SLM2, and, **c**, the back aperture of the objective. Bottom, profiles of the x-y intensity distributions at SLM1 (solid blue line), SLM2 and the objective (dashed cyan lines and dashed-dotted blue line indicates x and y-direction, respectively). Red dashed and dashed-dotted lines indicate the lateral edges of SLM1, SLM2 and the objective back aperture in the x and y direction, respectively. (**d**) Calculated light distribution at SLM2 as described by Equation 15 (blue-dashed line) and Equation 16 (green line) described in Supplementary Note 2. (**e**) Light distribution passing through the objective. Red curve represents experimental data and dashed black curve represents the theoretical curve described by Equation 20, in Supplementary Note 2.

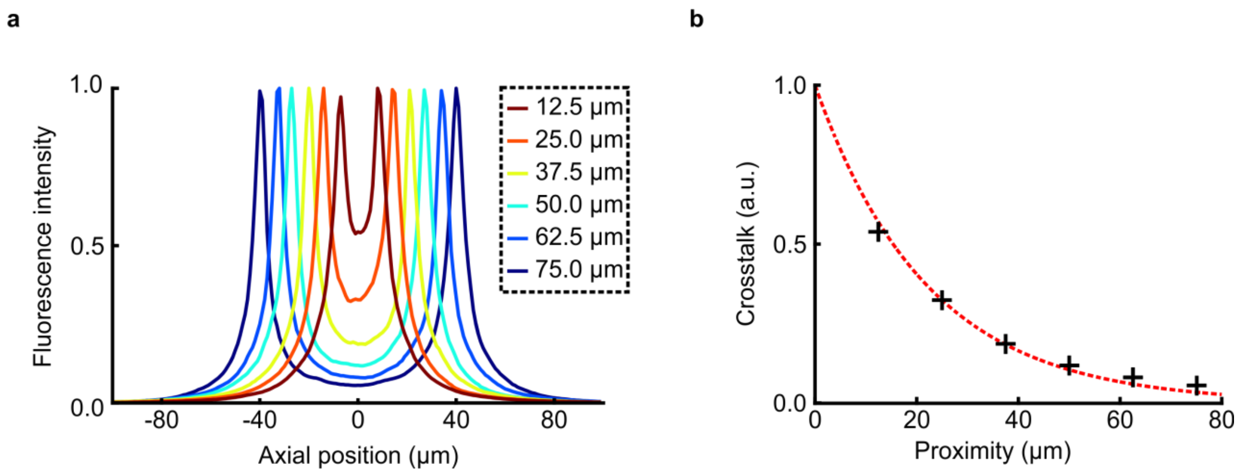

Supplementary Figure 8 │ 3D-CGH-TF: Characterization of intensity artifacts between two proximal planes. (**a**) Axial profile of the integrated fluorescence intensity on the area covering two 20-μm-diameter holographic spots having same lateral position but lying in two planes separated by a variable distance (inset). (**b**) Normalized crosstalk fluorescence intensity versus distance between the two planes. We notice that for spots projected in planes separated by 15 μm, crosstalk fluorescence can reach 50%. Depending on the TF properties of the system (achievable axial confinement), this distance may also vary with the size of the spot. In our system it should be the same for a large range of spot sizes (Supplementary Fig. 1c-d).

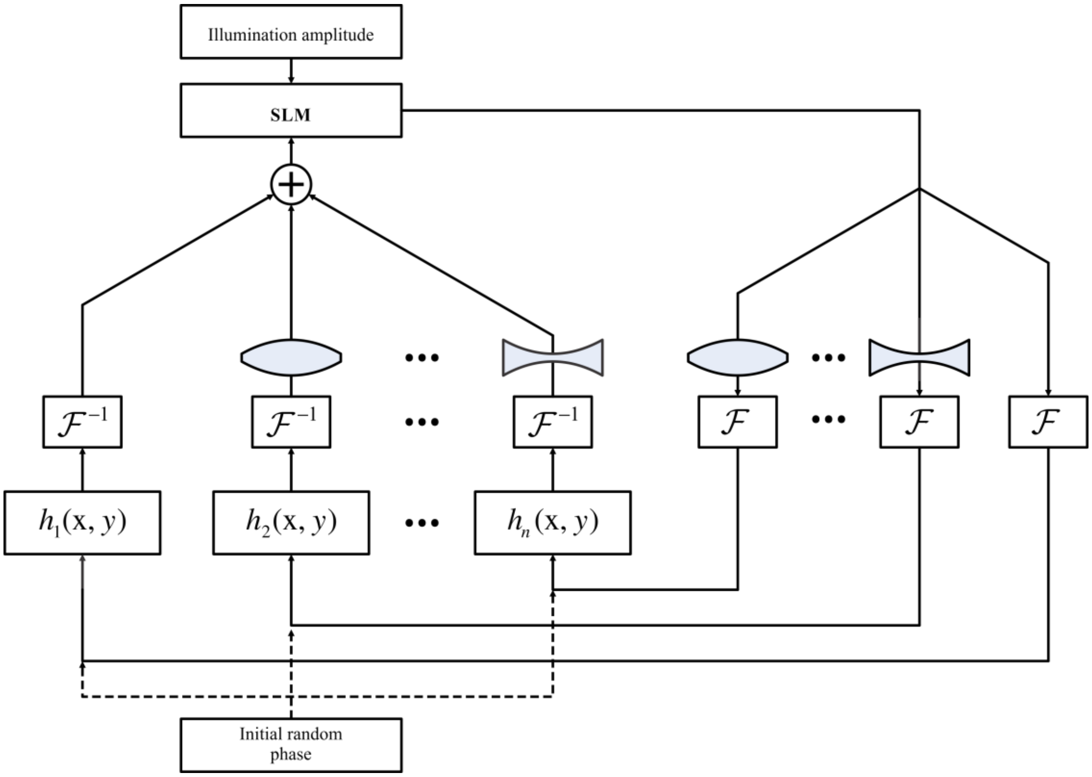

Supplementary Figure 9 │ Multi-plane Gerchberg and Saxton algorithm. Schematic diagram of the Gerchberg and Saxton algorithm for multi-plane pattern projection adapted from Haist et al.[3]. For each axial target two lens-phase functions of equal power and opposite sign are inserted into the Fourier-transform-based iterative process that relays the electric field distribution from the SLM to the sample plane. The first lens is added to the electric field distribution at the SLM plane and is used to control the axial position of the target in the sample volume. The second lens, of opposite sign, is added to the electric field distribution at the sample plane to cancel the effects of the first lens. This enables to project all targets into a single plane before the successive iteration. Under this condition, back and forth propagation of the electric field, $h_{\mathrm{n}}(x, y)$, converges to a phase profile that enables light shaping in multiple axial planes.

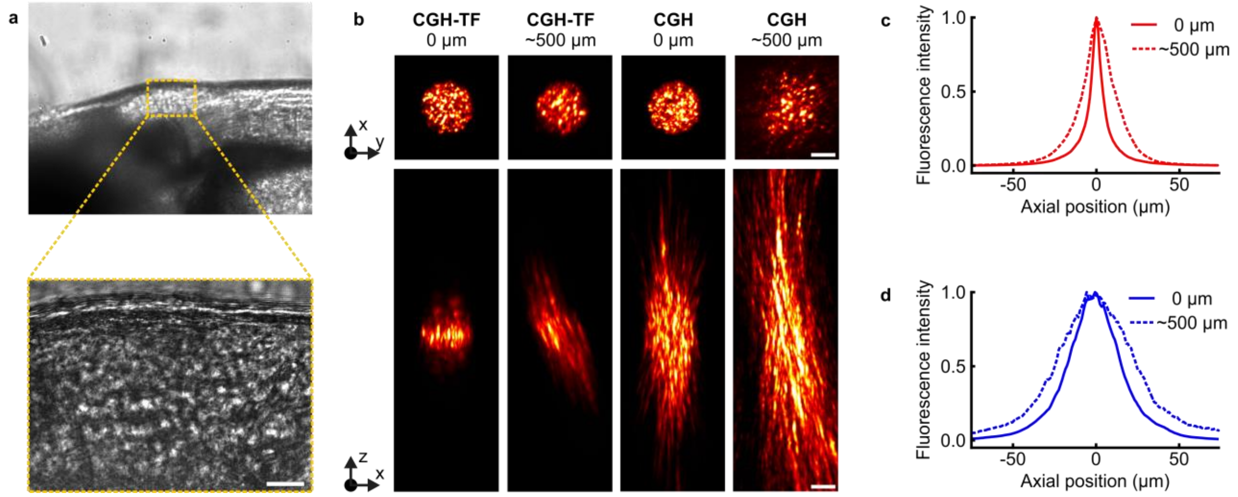

Supplementary Figure 10 | Propagation of CGH spots in the zebrafish brain. (**a**) Brightfield image of zebrafish larva brain, with a zoom in the area where we photoconverted neurons (bottom; see also area indicated in dark yellow box in Fig. 5a). Scale bar: 20 µm. To characterize the effect of light scattering of the specific sample we propagated CGH spots, with or without TF, through the brain. The fish was mounted on the lateral side facing up, as shown in Fig. 5a, and the laser beam propagated through the brain in the orthogonal direction (~500 µm). The larva, anesthetized and embedded in agarose, was put on top of a glass coverslip spin-coated with a thin film of Rhodamine-6G. CGH spots passing through the brain excited 2P fluorescence that was collected and imaged through OBJ2 on the CCD camera (see experimental setup in Fig.1). (**b**) Top panels, from left to right: 2P excited fluorescence from a CGH-TF spot without scattering (0 µm of tissue) and after going through the brain (~500 µm of tissue), and from a CGH spot without scattering and after going through the brain. The numbers on top of the panels correspond to the thickness of the zebrafish tissue between the objective and the Rhodamine layer. Scale bars: 10 µm. Bottom panels: Corresponding x-z cross section of the 2P excited fluorescence for each case presented in the top panels. z-stacks were recorded by imaging a fixed plane with OBJ2, while scanning the excitation objective, OBJ1. (**c**) 2P fluorescence intensity distribution around the focal plane of OBJ1 (z = 0 µm) for a CGH-TF spot with (dashed curve) and without scattering (solid curve). The axial resolution degrades from 7.8 ± 0.3 µm without scattering to 23.2 ± 2.7 µm with scattering (FWHM values; mean ± s.d. propagation measurements repeated in 4 different locations). (**d**) Same as in **c** for a CGH spot with (dashed curve) and without scattering (solid curve). In this case the axial resolution changes from 33.7 ± 1.0 µm without scattering to 43.7 ± 5.7 µm with scattering (FWHM values; mean ± s.d. propagation measurements repeated in 4 different locations). Transmission of laser light intensity after the tissue was about 25%.

In agreement with experiments on rat brain[4,5], these results revealed that although axial confinement of CGH-TF beam deteriorates with depth, still remains well below what is achievable with CGH alone. The tilt visible in the lateral view of axial propagations was already observed for shaped beam propagating through mice brain[4,5], and is probably due to the refractive index mismatch in the surface of the tissue[6].

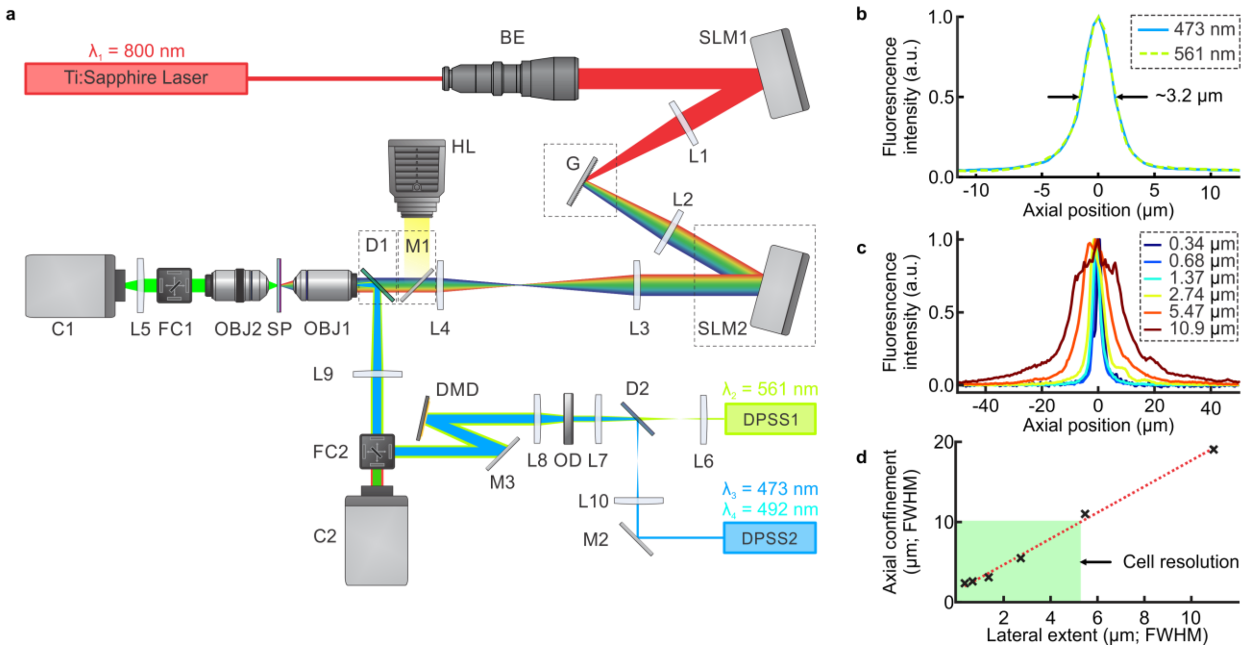

Supplementary Figure 11 │ 3D-CGH-TF setup combined with HiLo/DMD imaging. (**a**) The optical system for CGH and TF was combined with a two-color HiLo imaging setup to better resolve fluorescent neurons expressing Kaede in zebrafish, and with a digital micromirror device (DMD) for multipoint calcium imaging. Continuous wave (CW) diode pumped solid state lasers (DPSS) at 473 nm and 561 nm were used to excite green and red Kaede fluorescence state, respectively. For the optogenetics experiment described in Supplementary Note 3 the 473-nm laser was replaced with a 491-nm one, for reducing ChR2 excitation during calcium imaging. In both cases, the two laser beams joined the same optical path on D2 (dichroic mirror) and were collimated through lenses L6, L7 and L10 on an oscillating diffuser (OD) that was imaged through lenses L8 and L9 at the back aperture of the excitation objective OBJ1. For HiLo imaging the DMD was set in wide-field illumination mode (i.e. with all the micromirrors in the 'on' state), while for calcium imaging the OD was in steady state (i.e. not oscillating). The laser beams joined the excitation path on D1 (dichroic mirror), which also reflects collected fluorescence to a CMOS camera (C2) through the appropriate filter cube (FC2) for green or red fluorescence. For HiLo microscopy two fluorescence images are acquired for each plane: one with the OD in steady state, which produces a speckle illumination, and one with the OD in motion, in which the speckle pattern is smoothed out producing a uniform illumination image. A halogen lamp (HL) was used to perform transmitted light imaging of the sample through a removable mirror (M1) that helped on identifying the different regions of interest. Transmission images were recorded on a CCD camera (C1) through OBJ2. (**b**) Normalized fluorescence intensity versus axial position indicating the axial resolution (FWHM) of the HiLo imaging system (green curve for green fluorescence, cyan curve for red fluorescence). (**c**) Normalized fluorescence intensity versus axial position indicating the axial resolution (FWHM) of the DMD system for different spot sizes shown in the inset. The pixel size of the DMD at the sample plane was 0.34 µm. (**d**) Plot of the axial confinement (FWHM) of the DMD system for the different spot sizes shown in (**c**). The green rectangle indicates a typical cell size.

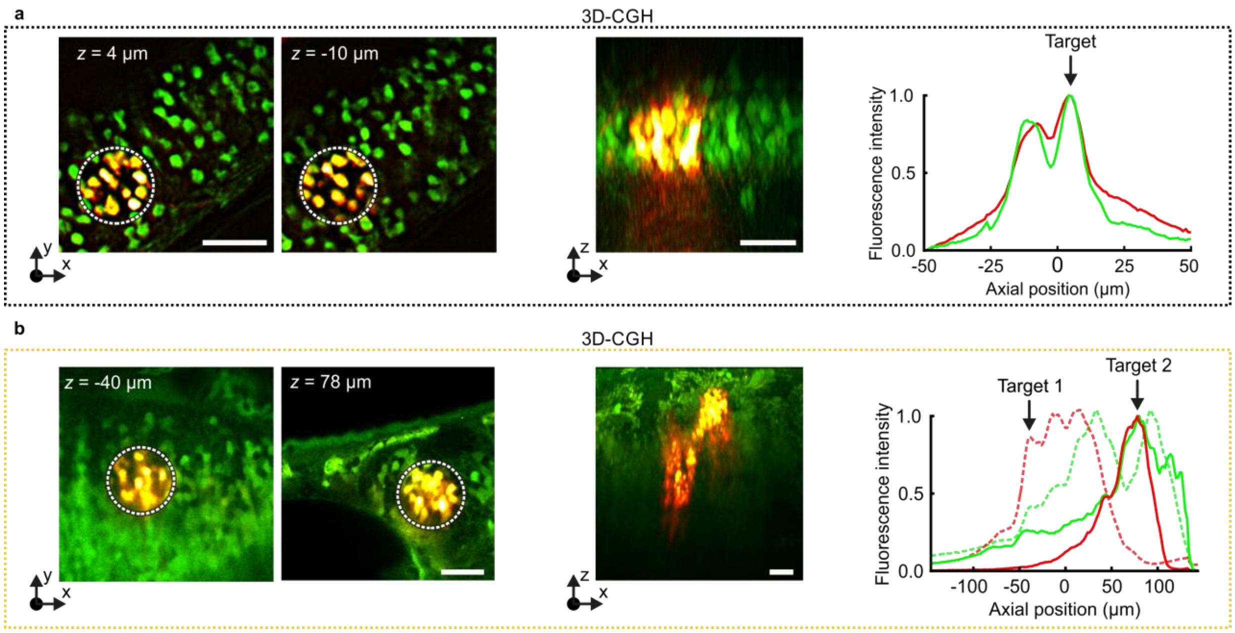

Supplementary Figure 12 │ 2P neural-ensemble photoconversion with 3D-CGH. (**a**) Left, overlaid HiLo green-pre- and red-post-photoconversion fluorescence images of Kaede-expressing neurons in the zebrafish spinal cord. We used one 35-µm-diameter holographic spot to photoconvert a neural ensemble (0.04 mW μm^{-2}, 200 pulses of 50 ms). The spot was focused at the upper Kaede-expressing layer (z = 4 µm). However, merged HiLo images showed significant photoconversion levels also in the lower layer (z = -10 µm). This is also evident in the middle panel where we show the orthogonal maximum fluorescence intensity projection of overlaid HiLo pre- and post-photoconversion images (green and red fluorescence respectively) and in the graph on the right where we show the axial distribution of green pre- and red post-photoconversion integrated fluorescence intensity. Scale bars: 30 µm. (**b**) Left, overlaid 2P green-pre- and red-post-photoconversion fluorescence images of Kaede-expressing neurons in the brain. Two 35-µm-diameter holographic spots photoconverted neurons at z = -40 µm and z = 78 µm (0.07 mW μm^{-2}, 8000 pulses of 50 ms). Right, axial distributions of green pre- and red post-photoconversion integrated fluorescence intensity for the two planes. The axial position of each target spot is indicated. Red fluorescence is less specific than in the case of 3D-CGH-TF spots of the same size (Fig. 5e). Scale bars: 30 µm, λ_{phot} = 800 nm. Axial distances are measured from the focal plane of the objective, which was focused at about 150 µm from the surface (defined as the beginning of green fluorescence).

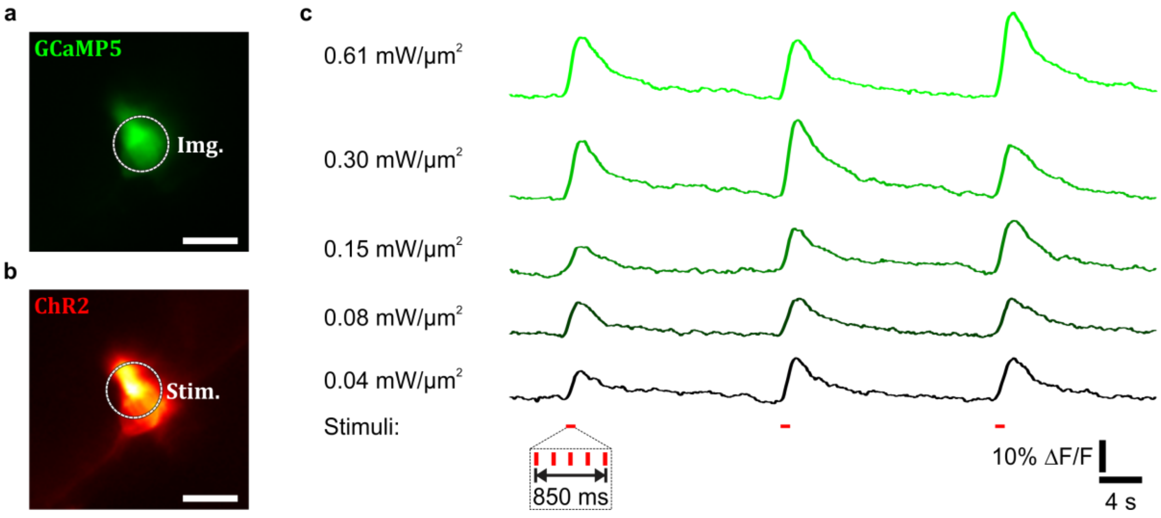

Supplementary Figure 13 | Power spectrum for optogenetic activation of a neuron expressing ChR2-mCherry and GCaMP5G in the *Tg(pkd2l1:gal4;UAS:ChR2-H134R-mCherry;UAS:GCaMP5G)*. (**a**) Green fluorescence of a spinal-cord neuron expressing GCaMP5G and (**b**) red fluorescence of the same neuron expressing also ChR2-mCherry. The circles on the cells indicate the position of the DMD illumination spot for imaging (**a**) and the position of the holographic spot for stimulation (**b**). In both cases the spot diameter was 10 μm. Scale bars: 10 μm; λ_{excit} = 900 nm. (**c**) Calcium imaging traces recorded by stimulating the neuron in **a** or **b** with different excitation power densities (0.04, 0.08, 0.15, 0.30 and 0.61 mW μm^{-2}, which corresponded to 3, 6, 12, 24 and 48 mW per spot at the sample plane). The power of the 491 nm laser used for calcium imaging was 18.6 - 37.8 μW mm^{-2}.

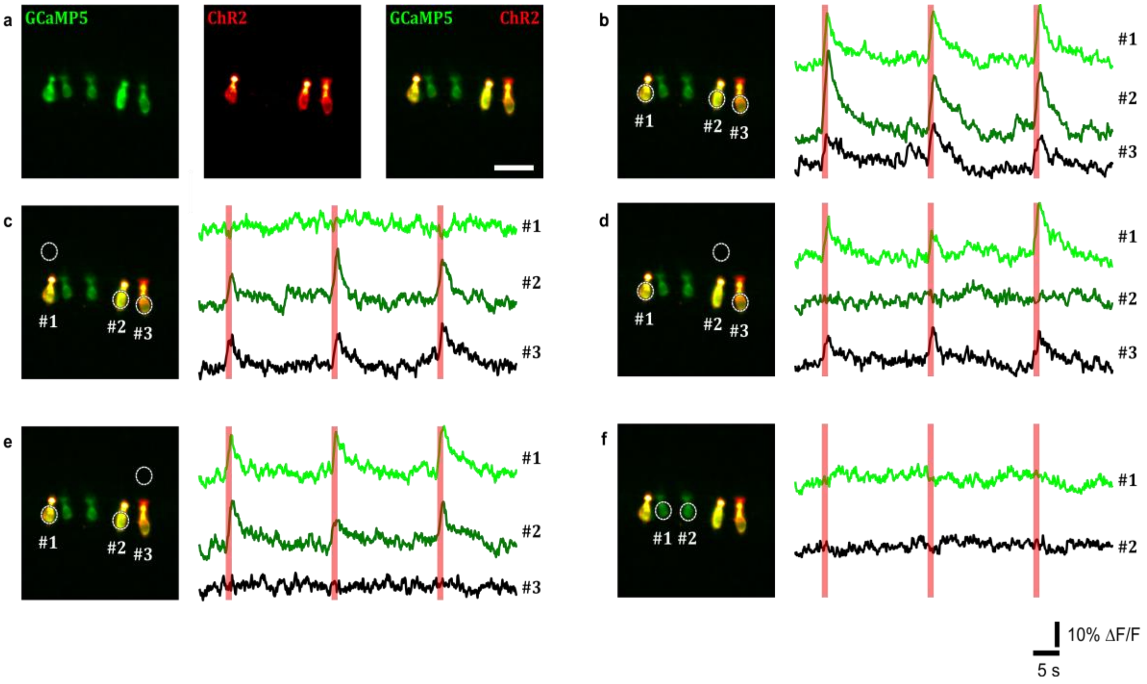

Supplementary Figure 14 | Multi-cell optogenetic activation of neurons co-expressing ChR2-H134R-mCherry and GCaMP5G. (**a**) Green and red fluorescence images in the *Tg(pkd2l1:gal4; UAS:ChR2-H134R-mCherry; UAS:GCaMP5G)* reveals that most but not all neurons co-expressed the two proteins. Scale bar: 25 μm. (**b**) Simultaneous stimulation of the 3 positive cells with 3 CGH spots of 10 μm in diameter. All cells efficiently responded to stimulation (0.61 mW μm^{-2}). (**c-e**) Spatial selectivity experiment: each time one spot was moved out of the target cell. The cell that was not stimulated did not respond. (**f**) Control experiment: photostimulating the two ChR2-negative cells did not lead to any responses. Vertical red lines indicate infrared laser stimulation. λ_{excit} = 900 nm; imaging laser power density: ~30.8 μW mm^{-2}.

SUPPLEMENTARY NOTE 1: EXCITATION VOLUME

In CGH the LCOS-SLM pitch-size at the objective back focal plane sets, for a given wavelength, the maximum accessible volume for illumination and induces a position-dependent diffraction efficiency (defined as the ratio between the light illuminating the LCOS-SLM and the light diffracted in the holographic pattern)[7,8]. As described by Golan et al.[7], the maximum displacement (or the Field Of Excitation) can be defined as the maximum aliasing-free displacement, i.e., when the sampling of the phase profiles satisfies the Nyquist theorem.

Lateral displacement limit

The phase profile required for a given lateral displacement, x_0, is

$$\phi(x) = \frac{k}{f_{\mathrm{eq}_1}} x_0 x \quad (1),$$

where k is the wavenumber and $f_{\mathrm{eq}_1} = \frac{f_1 f_3 f_{\mathrm{obj}}}{f_2 f_4}$ is an equivalent focal length taking into account all the intermediate lenses of the telescopes. Imposing the aliasing-free condition[9], we obtain

$$\frac{\delta\phi(x)}{\delta x} = \frac{k}{f_{\mathrm{eq}_1}} x \leq \frac{\pi}{a} \quad (2),$$

where a is the pixel pitch. Consequently, the maximum lateral displacement is

$$|\, x_{\mathrm{max}}| = \frac{\pi f_{\mathrm{eq}_1}}{ka} \quad (3),$$

which in our case leads to a field of excitation: $FOE = 2|x_{\mathrm{max}}|$=240 µm.

Axial displacement limit

Within the approximation of small defocus and high numerical aperture, the phase profile required for a given axial displacement, Δz, in polar coordinates describes a spherical function

$$\phi(r) = k\, \Delta z \sqrt{n^2 - \frac{r^2}{f_{\mathrm{eq}_2}^2}} \quad (4),$$

where n is the index of refraction of the immersion medium, Δz is the axial displacement and $f_{\mathrm{eq}_2} = \frac{f_3 f_{\mathrm{obj}}}{f_4}$.

Following the same criteria as above,

$$\frac{\delta\phi(r)}{\delta r} = -\frac{r\, k\, \Delta z}{f_{\mathrm{eq}_2}\sqrt{n^2 f_{\mathrm{eq}_2}^2 - r^2}} \leq \frac{\pi}{a} \quad (5).$$

The projection of the objective back aperture onto the SLM plane has a maximum radius of

$$r_{\mathrm{max}} = \frac{D f_3}{2 f_4} = NA\, f_{\mathrm{eq}_2} \quad (6),$$

where D is the diameter of the back aperture and NA is the numerical aperture of the objective. Hence, the maximum axial displacement without aliasing is given by

$$|\Delta z_{\mathrm{max}}| = \frac{\pi f_{\mathrm{eq}_2}\sqrt{n^2 - \mathrm{NA}^2}}{a\, k\, \mathrm{NA}} \quad (7)$$

and in our case leads to a field of excitation: $FOE_{\mathrm{z}} = 2|\Delta z_{\mathrm{max}}|$=261.13 µm.

In theory, we can simultaneously project several patterns, of different size and shape, up to fill this entire excitation volume. In practice, available laser power and possible intensity crosstalk in between the planes due to proximity of the patterns (see for instance Fig. 4b-c), limit the maximum number of spots that can be generated in each plane.

SUPPLEMENTARY NOTE 2: LIGHT DISTRIBUTION IN A 3D-CGH-TF SYSTEM

In order to optimize the optical design for 3D-CGH-TF, is important to derive an expression to estimate the light distribution at the objective back focal plane and therefore the amount of light transmitted through the objective. To this end, let us first consider the propagation of a Gaussian beam having at the SLM1 plane a waist of 2σ and a spectral bandwidth σ_λ, and let us follow the propagation of the central wavelength, λ_0. Assuming a flat wavefront, the electric field distribution for the x-axis (orthogonal to the grating lines) of the beam illuminating SLM1 can be described by:

$$U^-_{\mathrm{SLM1_x}}(x_1) = \frac{1}{\sqrt{2\pi}\sigma} e^{-\frac{1}{4}\left(\frac{x_1}{\sigma}\right)^2} \quad (8)$$

and after being cropped by the aperture of the SLM:

$$U^+_{\mathrm{SLM1_x}}(x_1) = U^-_{\mathrm{SLM1}}(x_1)\, \Pi(x/\Delta x_{\mathrm{SLM1}}) \quad (9),$$

where $\Pi(x) = \begin{cases} 0 & if\ |x| > 1/2 \\ 1/2 & if\ |x| = 1/2 \\ 1 & if\ |x| < 1/2 \end{cases}$.

The first lens (L1; f_1) Fourier transforms the electric field

$$U^-_{\mathrm{G_x}}(x_2) = \frac{1}{\sqrt{\lambda f_1}} \int_{-\infty}^{\infty} U^+_{\mathrm{SLM1}}(x_1) e^{-j\frac{2\pi}{\lambda f_1}x_1 x_2} dx_1 \quad (10),$$

which impinges on the grating at an incidence angle θ_I. Because of the oblique incidence, the electric field is stretched horizontally:

$$U^+_{\mathrm{G_x}}(x_2) = \frac{1}{|\cos(\theta_\mathrm{I})|} U^-_{\mathrm{G}}(\cos(\theta_\mathrm{I}) x_2) \quad (11).$$

The second lens (L2; f_2) Fourier transforms the electric field onto the second SLM (SLM2) plane:

$$\begin{aligned}
U^-_{\mathrm{SLM2_x}}(x_3) &= \frac{1}{\sqrt{\lambda f_2}} \int_{-\infty}^{\infty} U^+_{\mathrm{G}}(x_2) e^{-j\frac{2\pi}{\lambda f_2}x_2 x_3} dx_2 \\
&= \frac{1}{\sqrt{\lambda f_2}} \int_{-\infty}^{\infty} \frac{1}{|\cos(\theta_\mathrm{I})|} U^-_{\mathrm{G}}(\cos(\theta_\mathrm{I})\, x_2) e^{-j\frac{2\pi}{\lambda f_2}x_2 x_3} dx_2 \\
&= \frac{1}{\sqrt{\lambda f_2}} \int_{-\infty}^{\infty} \frac{1}{|\cos(\theta_\mathrm{I})|} \frac{1}{\sqrt{\lambda f_1}} \int_{-\infty}^{\infty} U^+_{\mathrm{SLM1}}(x_1) e^{-j\frac{2\pi}{\lambda f_1}x_1 x_2 \cos(\theta_\mathrm{I})} dx_1\, e^{-j\frac{2\pi}{\lambda f_2}x_2 x_3} dx_2 \\
&= \frac{1}{|\cos(\theta_\mathrm{I})|} \frac{1}{\sqrt{\lambda f_1}} \frac{1}{\sqrt{\lambda f_2}} \int_{-\infty}^{\infty} \int_{-\infty}^{\infty} U^+_{\mathrm{SLM1}}(x_1) e^{-j\frac{2\pi}{\lambda f_1}x_1 x_2 \cos(\theta_\mathrm{I})} dx_1\, e^{-j\frac{2\pi}{\lambda f_2}x_2 x_3} dx_2 \quad (12). \\
&= \frac{1}{|\cos(\theta_\mathrm{I})|} \frac{1}{\lambda\sqrt{f_1 f_2}} \int_{-\infty}^{\infty} \int_{-\infty}^{\infty} U^+_{\mathrm{SLM1}}(x_1) e^{-j\frac{2\pi}{\lambda f_1}x_1 x_2 \cos(\theta_\mathrm{I})} dx_1\, e^{-j\frac{2\pi}{\lambda f_2}x_2 x_3} dx_2 \\
&= \frac{1}{|\cos(\theta_\mathrm{I})|} \frac{1}{\lambda\sqrt{f_1 f_2}} \int_{-\infty}^{\infty} U^+_{\mathrm{SLM1}}(x_1) \int_{-\infty}^{\infty} e^{-j\frac{2\pi}{\lambda f_1}x_1 x_2 \cos(\theta_\mathrm{I})}\, e^{-j\frac{2\pi}{\lambda f_2}x_2 x_3} dx_2 dx_1 \\
&= \frac{1}{|\cos(\theta_\mathrm{I})|} \frac{1}{\lambda\sqrt{f_1 f_2}} \int_{-\infty}^{\infty} U^+_{\mathrm{SLM1}}(x_1) \delta\left(\cos(\theta_\mathrm{I}) \frac{x_1}{\lambda f_1} + \frac{x_3}{\lambda f_2}\right) dx_1
\end{aligned}$$

Applying the general scaling property of the Dirac delta function, ($\delta(ax - x_0) = \delta(x - x_0/a)/|a|$), this equation reduces to:

$$U^-_{\mathrm{SLM2_x}}(x_3) = \frac{1}{\cos^2(\theta_\mathrm{I})} \sqrt{\frac{f_1}{f_2}}\, U^+_{\mathrm{SLM1}}\left(-\frac{1}{\cos(\theta_\mathrm{I})} \frac{f_1}{f_2} x_3\right) \quad (13).$$

The lateral stretching of the electric field at the grating plane translates into a proportional shrinking at the SLM2 plane. The grating linearly diffracts all spectral components that are then collimated by L2 and impinge on SLM2 laterally shifted from the optical axis by:

$$\Delta x \approx \frac{f_2}{g} \Delta\lambda \quad (14),$$

where *g* is the grating grooves' period and $\Delta\lambda = |\lambda_0 - \lambda|$.

The illumination intensity at SLM2 can be described as a convolution between the electric field distribution at the SLM2 plane for the central wavelength and the spectral distribution[10]:

$$
\begin{aligned}
I^{-}_{\mathrm{SLM2_x}}(x) &= \int_{-\infty}^{\infty} |U^{-}_{\mathrm{SLM2}}(x,\Delta\lambda)|^2 d\Delta\lambda \\
&= \left|U^{+}_{\mathrm{SLM1}}\left(-\frac{1}{cos(\theta_{\mathrm{I}})}\frac{f_1}{f_2}x\right)\right|^2 \otimes e^{-\frac{1}{2}\left(\frac{\Delta\lambda}{\sigma_\lambda}\right)^2} \\
&\simeq e^{-\frac{1}{2}\left(\frac{x}{\sigma_{\mathrm{x}}\, cos(\theta_{\mathrm{I}}) f_2/f_1}\right)^2} \Pi\left(\frac{x}{\Delta_{\mathrm{x_{SLM1}}}\, cos(\theta_{\mathrm{I}}) f_2/f_1}\right) \otimes e^{-\frac{1}{2}\frac{x^2}{\left(\frac{f_2\sigma_\lambda}{g}\right)^2}} \qquad (15), \\
&\simeq \int_{-c}^{c} e^{-a\tau^2 - b(\mathrm{x}-\tau)^2} d\tau \\
&\simeq e^{-\frac{abx^2}{a+b}}\left(\mathrm{erf}\left(\frac{(a+b)c-bx}{\sqrt{a+b}}\right) + \mathrm{erf}\left(\frac{(a+b)c+bx}{\sqrt{a+b}}\right)\right)
\end{aligned}
$$

where $a = \frac{1}{2}(\sigma_{\mathrm{x}}\cos(\theta_{\mathrm{I}}) f_2/f_1)^{-2}$, $b = \frac{1}{2}\left(\frac{f_2\sigma_\lambda}{g}\right)^{-2}$, $c = \frac{1}{2}\Delta_{\mathrm{x_{SLM1}}}\cos(\theta_{\mathrm{I}})\, f_2/f_1$ and τ is the dummy variable of the convolution. For simplicity, we have omitted the proportionality factors. This equation simplifies if we neglect the effect of the SLM1 aperture. Under this approximation $|x| \ll \frac{c}{b}(a+b)$, and one can derive from equation (15) the light distribution at SLM2 and therefore at the objective back aperture along the x-axis:

$$
I^{-}_{\mathrm{SLM2_x}}(x) = e^{-\frac{abx^2}{a+b}} \qquad (16).
$$

Equations (15) and (16) are plotted in Supplementary Fig. 7d (blue and green lines, respectively). For our experimental conditions, the discrepancy between these two curves is less than 1%.

Light distribution along the y-axis on SLM2 can be obtained in a similar way, but without having in that case any shrinking coefficient or linear dispersion by the grating:

$$
I^{-}_{\mathrm{SLM2_y}}(y) = e^{-\frac{1}{2}\left(\frac{f_1}{f_2}\frac{y}{\sigma}\right)^2} \qquad (17).
$$

The y-light distribution is also plotted in Supplementary Fig. 7.

We can also derive the analytical expression for the FWHM of the illumination distribution along the x- and y-axis:

$$
\begin{aligned}
\sigma_{\mathrm{x_{BFP}}} &= \frac{f_4}{f_3}\sqrt{\frac{2\sigma^2\cos(\theta_{\mathrm{I}})^2 f_2^2}{f_1^2} + \frac{2f_2^2\sigma_\lambda^2}{g^2}} \\
\sigma_{\mathrm{y_{BFP}}} &= \frac{f_2}{f_1}\frac{f_4}{f_3}\sigma
\end{aligned} \qquad (18).
$$

The same formalism can be applied to derive the analytical expression for the FWHM of the illumination distribution along the x- and y-axis for a conventional TF system, i.e. with a collimated Gaussian beam illuminating the TF grating. This can be done by taking for the illumination of SLM1 a Gaussian beam with an initial small beam waist. For instance, a Gaussian beam of approximately 300 µm in diameter at SLM1 will generate a 10-µm-diameter spot at the sample plane. In this limit $\frac{2\sigma^2\cos(\theta_{\mathrm{I}})^2 f_2^2}{f_1^2} \ll \frac{2f_2^2\sigma_\lambda^2}{g^2}$ and equations (18) become:

$$
\sigma_{\mathrm{x_{BFP}}} = \sqrt{2}\frac{f_4}{f_3}\frac{f_2}{d}\sigma_\lambda \qquad (19).
$$

$$\sigma_{\mathrm{y_{BFP}}} \quad = \quad \frac{f_2}{f_1}\frac{f_4}{f_3}\sigma$$

Equations (18) and (19) can be particularly practical for designing temporal focusing experimental setups.

Finally, in order to properly resize hologram tiles and produce equal intensity patterns, we calculate the energy distribution passing through the objective as a function of the vertical position:

$$\begin{aligned} I_{\mathrm{BFP}}^{+}(y) &= \int_{-\sqrt{(D/2)^2-y^2}}^{\sqrt{(D/2)^2-y^2}} I_{\mathrm{SLM2}}^{+}\left(\frac{f_4}{f_3}x\right) I_{\mathrm{SLM2}}^{+}\left(\frac{f_4}{f_3}y\right) dx \\ &= \int_{-\sqrt{(D/2)^2-y^2}}^{\sqrt{(D/2)^2-y^2}} e^{-\frac{ab}{a+b}\left(\frac{f_3}{f_4}\right)^{1/2} x^2 - d\,y^2} dx \qquad (20), \\ &= e^{-d\,y^2}\,\mathrm{erf}\left(\sqrt{\frac{1}{2}\frac{ab}{a+b}\left(\frac{f_3}{f_4}\right)^{\frac{1}{2}}\left[\left(\frac{D}{2}\right)^2 - y^2\right]}\right)^2 \end{aligned}$$

where $d = \frac{1}{2}\left(\frac{\sigma_{\mathrm{xy}} f_2 f_4}{f_1 f_3}\right)^{-2}$ and D represents the aperture of the objective. This distribution was experimentally verified and the results are plotted in Supplementary Fig. 7e. The size of the vertical tiles of the SLM for each plane was chosen by taking into account this distribution, the diffraction efficiency (Supplementary Fig. 3) and the total area of each holographic pattern.

SUPPLEMENTARY NOTE 3: OPTOGENETIC ACTIVATION OF NEURONS IN THE SPINAL CORD OF ZEBRAFISH LARVAE

In this section we test the illumination conditions (excitation power and exposure time) enabling optogenetic photostimulation in the spinal cord of zebrafish. Holographic illumination was used to photoactivate neurons expressing both ChR2 and GCaMP5G *Tg(pkd2l1:gal4; UAS:ChR2-H134R-mCherry; UAS:GCaMP5G*). For imaging the fluorescence changes induced to GCaMP5 by neuronal activation, we used the system described in Supplementary Fig. 11. In order to reduce excitation of ChR2 while performing calcium imaging we used a laser source emitting at 491 nm (Cobolt, Calypso) and patterned illumination through a digital micromirror device (DMD). As previously demonstrated in mice brain[11], patterned illumination into multiple small regions instead of widefield calcium imaging enables concentrating light illumination selectively on the target, with a drastic reduction of ChR2-activation during imaging. In addition, the presence of the diffuser after the DMD enabled scrambling the beam phase and achieving an axial confinement below 10 μm for illumination areas close to the cell size (Supplementary Fig. 11c-d). Finally, the recording was synchronized with a mechanical shutter enabling to record calcium transients between optogenetic stimulation pulses[11] (Supplementary Fig. 13).

We performed two sets of experiments: a first one to assess the illumination parameters to achieve reliable neuronal activation (Supplementary Fig. 13), and a second one to demonstrate spatial selectivity (Supplementary Fig. 14) when multiple cells are targeted.

In the first set of experiments, we used a 10-μm-diameter holographic spot (λ_{excit} = 900 nm) and a stimulation protocol comprising 5 pulses of 50 ms each and 150 ms interval time, repeated three times with stimulation density ranging from 0.04 mW μm^{-2} to ~0.6 mW μm^{-2}. Out of 5 cells co-expressing ChR2 and GCaMP5G, we were able to record calcium signals of 8.4 ± 4.5 % ΔF/F (n=5) using stimulation power densities as low as ~0.04 mW μm^{-2} (corresponding to 3 mW per spot at the sample plane). For a power density of 0.08 mW μm^{-2} (6 mW per spot in the sample plane), we detected calcium responses reaching approximately 10% ΔF/F. The calcium signals were analyzed as described in the work by Jia et al.[12]

On a second set of experiments, we took advantage of the light shaping capabilities of the system to perform simultaneous stimulation of multiple cells (Supplementary Fig. 14). The low co-expression of both proteins limited the number of cells that could be excited within the same FOV, which were also confined within a few micrometers near the central canal of the zebrafish spinal cord (Supplementary Fig. 14a). Moreover, the expression level for both the opsin and the calcium indicator varied significantly from cell to cell leading to distinct levels of fluorescence for the same imaging power (Supplementary Fig. 14b), as well as different neural activity responses to the same stimulation power density (Supplementary Fig. 14c). Despite sample heterogeneity, our system allowed us to selectively and simultaneously control and monitor the neural activity of multiple cells (Supplementary Fig. 14c-g). The stimulation protocol used was the same as for experiment in Supplementary Fig. 13.

SUPPLEMENTARY REFERENCES

1. Gerchberg, R. W. & Saxton, W. O. A pratical algorithm for the determination of the phase from image and diffraction pictures. *Optik (Stuttg).* **35,** 237–246 (1972).
2. Persson, M., Engström, D. & Goksör, M. Real-time generation of fully optimized holograms for optical trapping applications. *Proc SPIE* **8097,** 80971H–80971H–9 (2011).
3. Haist, T., Schönleber, M. & Tiziani, H. . Computer-generated holograms from 3D-objects written on twisted-nematic liquid crystal displays. *Optics Communications* **140,** 299–308 (1997).
4. Bègue, A. *et al.* Two-photon excitation in scattering media by spatiotemporally shaped beams and their application in optogenetic stimulation. *Biomed. Opt. Express* **4,** 2869–2879 (2013).
5. Papagiakoumou, E. *et al.* Functional patterned multiphoton excitation deep inside scattering tissue. *Nat. Photonics* **7,** 274–278 (2013).
6. Chaigneau, E., Wright, A. J., Poland, S. P., Girkin, J. M. & Silver, R. A. Impact of wavefront distortion and scattering on 2-photon microscopy in mammalian brain tissue. *Opt. Express* **19,** 22755–22774 (2011).
7. Golan, L., Reutsky, I., Farah, N. & Shoham, S. Design and characteristics of holographic neural photo-

stimulation systems. *J. Neural Eng.* **6,** 66004 (2009).
8. Yang, S. *et al.* Three-dimensional holographic photostimulation of the dendritic arbor. *J. Neural Eng.* **8,** 46002 (2011).
9. Carcolé, E., Campos, J. & Bosch, S. Diffraction theory of Fresnel lenses encoded in low-resolution devices. *Appl. Opt.* **33,** 162–174 (1994).
10. Agrawal, G. P. Spectrum-induced changes in diffraction of pulsed optical beams. *Optics Commun.* **157,** 52–56 (1998).
11. Szabo, V., Ventalon, C., De Sars, V., Bradley, J. & Emiliani, V. Spatially Selective Holographic Photoactivation and Functional Fluorescence Imaging in Freely Behaving Mice with a Fiberscope. *Neuron* **84,** 1157–1169 (2014).
12. Jia, H., Rochefort, N. L., Chen, X. & Konnerth, A. In vivo two-photon imaging of sensory-evoked dendritic calcium signals in cortical neurons. *Nat. Protoc.* **6,** 28–35 (2011).
13. Klapoetke, N. C. *et al.* Independent optical excitation of distinct neural populations. *Nat. Methods* **11,** 338–346 (2014).
14. Lin, J. Y., Knutsen, P. M., Muller, A., Kleinfeld, D. & Tsien, R. Y. ReaChR: a red-shifted variant of channelrhodopsin enables deep transcranial optogenetic excitation. *Nat. Neurosci.* **16,** 1499–1508 (2013).
